# Chiral Analysis of Pesticides and Emerging Contaminants by Capillary Electrophoresis—Application to Toxicity Evaluation

**DOI:** 10.3390/toxics12030185

**Published:** 2024-02-28

**Authors:** Laura García-Cansino, María Luisa Marina, María Ángeles García

**Affiliations:** 1 Universidad de Alcalá, Departamento de Química Analítica, Química Física e Ingeniería Química, Ctra. Madrid-Barcelona Km. 33.600, 28871 Alcalá de Henares, Madrid, Spain; laura.garciacansino@uah.es (L.G.-C.); mluisa.marina@uah.es (M.L.M.); 2 Universidad de Alcalá, Instituto de Investigación Química Andrés M. del Río, Ctra. Madrid-Barcelona Km. 33.600, 28871 Alcalá de Henares, Madrid, Spain

**Keywords:** agrochemical formulations, capillary electrophoresis, chiral analysis, emerging contaminants, environmentally friendly analytical methodologies, food, pesticides, soil, toxicity, water

## Abstract

Chiral analysis is a very relevant topic in environmental chemistry. This is due to the different properties of the stereoisomers of chiral compounds. In the case of agrochemicals, the desired activity, degradation rate, or toxicity, among other characteristics, may differ between stereoisomers, and the same is true for emerging contaminants, such as pharmaceuticals and cosmetics. Therefore, the development of chiral analytical methodologies enabling their determination in samples of environmental interest is paramount. Although other techniques have been widely employed to carry out chiral separations, such as HPLC, GC, and SFC, capillary electrophoresis (CE) has attracted a lot of attention in the field of chiral analysis due to its simplicity, flexibility, and low cost. In fact, chromatographic columns are not needed, and the consumption of reagents and samples is very low due to the small dimensions of the separation capillaries. This article reviews the characteristics of the chiral methodologies developed by CE for the stereoselective analysis of pesticides and emerging contaminants in environmental samples (water and soil), as well as pesticides in food samples and commercial agrochemical formulations. Applications of the developed CE methodologies in stability and toxicity studies of these chiral contaminants are also reviewed.

## 1. Introduction

Chirality currently has a big impact on numerous fields such as the pharmaceutical, clinical, cosmetic, food, and environmental industries, among others. This is due to the different properties of the stereoisomers of chiral compounds. Approximately 30% of pesticides [1] and 60% of drugs [2,3] are chiral. Thus, stereoselective analyses of pesticides, drugs, and cosmetics in environmental samples by means of separation techniques have received increasing attention in recent years due to the different activities, degradation rates, and toxicities, among other properties, of the stereoisomers of these compounds [4]. In fact, one stereoisomer may be more active than another [5,6,7,8], have a different kind of biological activity [9,10], be inactive [11,12], or have a higher level of toxicity towards non-target organisms [5,13]. In cases in which enantiomers show different behaviors, toxicity data at the enantiomeric level are needed and are very useful in risk assessments and to promote the correct use of chiral agrochemicals [14]. Despite this, many agrochemicals are often used, regulated, and analyzed as racemic mixtures, partly due to insufficient knowledge of the factors that determine their possible enantioselective behavior, as well as the difficulties associated with the separation and analysis of enantiomers [14]. This represents an added environmental problem, since, in many cases, the fate of an enantiomer considered as inactive is unknown and may affect non-target organisms. For all these reasons, the correct assessment of environmental risk due to the presence of chiral pesticides and emerging chiral contaminants requires stability and toxicity studies at the enantiomeric level. In addition, it is important to carry out these studies with mixtures of different chiral pesticides and/or emerging chiral contaminants since they are not isolated in the environment and may have synergistic or antagonistic effects on each other.

In the case of agrochemicals, their widespread use makes their control in environmental and food samples necessary. Moreover, the quality control of commercial agrochemical formulations based on chiral pesticides is required, especially when they are marketed as pure stereoisomers to minimize their negative impact on the environment as well as their unwanted toxic effects on non-target organisms. It is known that 7% of chiral pesticides are marketed as pure or enriched stereoisomers of an active substance [15]. Regarding pharmaceuticals and cosmetics, their widespread use for hygienic purposes and disease treatment in humans and animals has resulted in their presence in environmental samples, mostly in water samples, and their consideration as emergent pollutants [16]. These emerging pollutants are generally found at very low concentrations in the environment. For example, concentrations at the µg L^−1^ level of anti-inflammatory and antihypertensive drugs have been found in wastewater matrices, as will be described later in this review. Anti-inflammatory drugs are widely employed, and therefore, they have been detected at high concentrations in urban wastewater [17]. For example, ibuprofen has been found to have the highest concentration, 40.8 µg L^−1^, in urban wastewater [17].

Although pharmaceuticals and cosmetics are recognized as emerging pollutants, their stereochemistry is very often ignored, and this has given rise to incorrect ecotoxicity, bioavailability, and accumulation data. Moreover, in general, toxicity parameters are calculated using initial concentrations of the pollutants and not their real concentrations in exposure tests. Several studies have recently been carried out to evaluate the stability and toxicity of the enantiomers of emerging chiral pollutants using their real concentrations as determined by CE [2,16,18,19,20,21]. In this sense, analytical separation techniques enable us to determine the concentration of stereoisomers in environmental samples and therefore to evaluate their individual stability and toxicity towards non-target organisms.

A sample pretreatment is usually necessary due to the complexity of environmental samples and the low concentrations at which analytes are present. Solid-phase extraction (SPE) or liquid–liquid extraction (LLE) are very frequently employed with this aim although other sample preparation techniques have also been used [22]. Regarding separation techniques, the most important ones for chiral analyses are gas chromatography (GC), high-performance liquid chromatography (HPLC), supercritical fluid chromatography (SFC), and capillary electrophoresis (CE) [23], with HPLC and CE being the most frequently used with this aim [3]. Chiral CE has been increasingly employed in the last few years due to some advantages, such as the following: its high level of efficiency [24], high availability of chiral selectors without the need to use stationary phases (minimal quantities of chiral selectors are employed [24,25]), high analysis speed [26], miniaturization [27], and low consumption of reagents and samples [28], with this last point being very important today in the context of green chemistry. The separation modes mainly used in CE to carry out chiral separations are electrokinetic chromatography (EKC) and capillary electrochromatography (CEC) [29]. EKC is the most used CE mode and consists of the addition of a “pseudophase” to the separation medium, which in the case of a chiral separation, must necessarily be a chiral selector that interacts enantioselectively with the analyte. Among all the chiral selectors used in EKC, such as cyclodextrins (CDs), macrocyclic antibiotics, proteins, chiral ionic liquids, polysaccharides, and chiral micelles, among others, CDs are undoubtedly the most widely employed due to their low ultraviolet (UV) absorbance and their high solubility in aqueous media, as well as their widespread availability [30]. Different EKC modes are defined depending on the nature of the chiral selector employed, for example, cyclodextrin electrokinetic chromatography (CD-EKC) [31] or micellar electrokinetic chromatography (MEKC) [32]. When the chiral compounds to be analyzed have a high level of hydrophobicity, it is common to combine micelles and cyclodextrins (CD-MEKC) [33] or use non-aqueous solvents (non-aqueous capillary electrophoresis, NACE) to increase the solubility. The wide variety of chiral selectors available on the market and the fact that it is not necessary to use a chiral column have favored the application of EKC to chiral analyses of contaminants. If the presence of a stationary phase in the capillary is considered convenient, the separation mode is called CEC, which is a hybrid technique between HPLC and CE [29].

This article reviews the advances achieved in the development of chiral methodologies by CE for the stereoselective analysis of pesticides and emerging contaminants (drugs and cosmetics) in environmental samples (water and soil) as well as for the chiral determination of pesticides in agrochemical formulations and food samples. The characteristics of the analytical methodologies developed are detailed and presented in tables, and their main applications are discussed, including those related to the evaluation of the stability and toxicity of enantiomers and racemic contaminants towards aquatic non-target organisms.

## 2. Chiral Analysis of Pesticides and Emerging Contaminants by CE

Table 1 groups the articles describing analyses of different classes of chiral pesticides (insecticides, herbicides, fungicides, and nematicides) in water, soil, food samples, and commercial formulations, as well as emerging chiral contaminants in water samples, including the characteristics of the chiral methodologies developed by CE. Among the different works reported, Table 1 shows that only two of them were based on the use of CEC as the separation mode using cellulose tris(3-chloro-4-methylphenylcarbamate) [34] or (+)-1-(4-aminobutyl)-(5R,8S,10R)-terguride as chiral stationary phases [35]. In one article, the separation mode chosen was NACE [36] while in the all other articles described, EKC was used as the separation mode. The articles are classified in this table according to the nature of the chiral compound analyzed. The works included in Table 1 reported the chiral separation of individual compounds as well as the simultaneous separation of mixtures of different compounds. The chiral selectors employed included CDs, the macrocyclic antibiotic vancomycin (VC), or bile salts. Mixtures of these chiral selectors were also employed and additives such as sodium dodecyl sulphate (SDS), urea, or organic solvents (acetonitrile (ACN) or methanol (MeOH)) were also present in some cases in the separation medium to facilitate or improve the chiral separations. CDs were used as the sole chiral selector in the separation medium as well as in dual systems of two CDs. The most used CDs were heptakis (2,3,6-tri-methyl)-β-cyclodextrin (TM-β-CD), γ-CD, 2-hydroxypropyl-β-cyclodextrin (HP-β-CD), and 2-hydroxypropyl-γ-cyclodextrin (HP-γ-CD). Considering the discrimination power of VC against anionic compounds with carboxylic groups, this compound was selected as the chiral selector in some of the articles reviewed [37,38,39]. Regarding the detection mode, UV detection was the most used method although fluorescence [40] was also employed, enabling researchers to improve the detection limits (LODs) at the µg L^−1^ level. Different techniques were used for sample treatment. Solid-liquid extraction (SLE) [34,35,36,38,41,42,43,44,45,46,47,48,49,50,51] or ultrasound-assisted extraction (UAE) [52] were used when the samples were solid. For the preconcentration of extracts obtained from solid samples or in the case of the extraction or preconcentration of liquid samples, SPE was the most frequently employed among the different techniques used [35,37,39,40,41,44,45,48,49,53,54,55,56,57,58,59,60], although the use of LLE [34,35,38,42,44,52] or pressurized-liquid extraction (PLE) [49] was also reported. In addition, the treatment of a liquid sample by on-line electrokinetic preconcentration was also described [61]. Regarding agrochemical formulations, a direct dilution in H_2_O [19,52], MeOH [43,46,47,62,63,64], in a separation buffer containing urea and sodium deoxycholate (SDC) [20], or in the background electrolyte (BGE)/H_2_O (50:50, *v*/*v*) [64] was carried out. The characteristics and applications of the methodologies developed for the chiral analysis of the compounds considered in this review are described in more detail as follows.

### 2.1. Insecticides

Insecticides are employed for the control of mosquitoes and ticks involved in the expansion of human and animal diseases [65]. They are widely used in numerous applications, such as agriculture and horticulture, among others, and constitute an important group of pesticides. As an example, approximately 10.4% of the pesticides used annually in Spain correspond to insecticides [66]. Depending on the chemical family, insecticides can be classified into different groups, such as organochlorines, organophosphorus, carbamates, pyrethroids, neonicotinoids, ryanoids, avermectins, and insecticide growth regulators (IGRs) [67]. Pyrethroid, organophosphorus, and sulfoximine insecticides have stereoselectively been separated and determined by CE in tap water, soil samples, and in commercial agrochemical formulations.

Pyrethroids are synthetic pesticides derived from natural pyrethrins which are modified to improve their biological activity and stability [68]. The pyrethroids that have been analyzed by CE are bifenthrin (BF) ((2-methyl-3-phenyl-phenyl)methyl-3-((Z)-2-chloro-3,3,3-trifluoro-prop-1-enyl)-2,2-dimethyl-cyclopropane-1-carboxylate) and tetramethrin ((1,3-dioxo-4,5,6,7-tetrahydroisoindol-2-yl)methyl 2,2-dimethyl-3-(2-methylprop-1-enyl)cyclopropane-1-carboxylate) which belong to the “fourth and second generation” of pyrethroids, respectively. Both are highly hydrophobic compounds (log Kow = 6.0 and 4.7, respectively) and difficult stereoselectively separate since they present two chiral centers in their structures and consequently, four stereoisomers. However, in the case of BF, only cis-BF is employed in commercial agrochemical formulations based on its higher insecticide activity with respect to trans-BF. Out of the two stereoisomers of cis-BF, 1R,cis-BF is the most toxic and persistent [69]. Taking into account that the insecticide activities of 1R,cis-BF and 1S,cis-BF are similar, the use of racemic cis-BF in commercial insecticide formulations is not justified. Enantiomerically pure commercial formulations based on 1S,cis-BF should be marketed, and adequate chiral methodologies with the potential to enable their quality control are required. In this context, cis-BF was enantiomerically separated by CD-MEKC, and due to its low solubility in water, the use of a separation medium consisting of sodium cholate (SC) (100 mM) as micellar system, TM-β-CD (20 mM), and 2 M urea as additives in a borate buffer (pH 8.0) was necessary [63]. An enantiomeric resolution (Rs) of 2.8 was achieved in 9.2 min. Cis-BF was enantiomerically quantified in a polyvalent commercial insecticide formulation marketed with racemic cis-BF and its results agreed with those indicated on the label. Although this formulation was commercialized as racemic cis-BF, the method showed great potential to be applied to the quality control of commercial formulations marketed as pure enantiomers. On the other hand, tetramethrin is found in a 80:20 proportion of trans:cis isomers, respectively, with the 1R enantiomer of the trans isomer being more active than the other isomers, followed by the 1S-cis isomer [70]. This pyrethroid was stereoselectively separated by CE using a dual system of chiral selectors formed by SDC and HP-β-CD in a borate buffer at a pH of 8.0 [20]. The baseline separation of the four stereoisomers was obtained in less than 12.5 min. This methodology was applied to the chiral analysis of a commercial antiparasitic formulation which also had to be dissolved in urea and SDC to increase its solubility. Percentages of 25% and 16% for trans and cis isomers, respectively, with respect to the labeled content, were obtained, showing the relevance of having stereoselective analytical tools to enable adequate quality control of these commercial agrochemical formulations.

In the 1950s, organophosphorus pesticides (OPs) were introduced for pest control in fruits, vegetables, and other crops as an alternative to chlorinated hydrocarbons, which persist in the environment. The asymmetric center in OPs is usually a phosphorus or carbon atom [71]. Although stereoselective (bio)discrimination has been frequently described for OPs, many of these pesticides are sold as racemates, then the chiral separation of OPs can be used to enantiomerically monitor the selective degradation of these racemates. Huang et al. carried out the individual enantiomeric separation of four neutral and poorly water-soluble OP pesticides (pyraclofos, profenofos, prothiofos, and sulprofos) by nonaqueous and aqueous-organic media [36]. NACE was applied for the pyraclofos enantioseparation based on the use of SC with SDS in a nonaqueous medium (MeOH/ACN (4:1, *v*/*v*)); however, SC and γ-CD were used as chiral selectors in an aqueous-organic medium (MeOH/H_2_O/ACN (5:4:1, *v*/*v*/*v*)) for the separation of the other three OPs, with the aim of increasing the low solubility of the CD in the presence of MeOH or ACN. Their analysis times ranged between 15 and 28 min, reaching resolution values around 2 min. The proposed method was applied to the determination of these chiral OPs in spiked soil samples after their extraction with MeOH. García-Ruiz et al. also investigated the individual chiral separation of a group of OPs (malathion, malaoxon, isomalathion, phenthoate, isophenphos, ruelene, phenamiphos, and naled) by CD-EKC with different anionic CDs as chiral selectors [54]. The use of a Tris (N-tris(hydroxymethyl) aminomethane) buffer at a pH of 7.0 and carboxymethyl-β-cyclodextrin (CM-β-CD) as a chiral selector made the individual separation of malathion and phenthoate enantiomers possible, as well as the partial chiral separation of phenamiphos and the separation of three (out of four) isomalathion enantiomers. However, the enantiomers of naled were separated with CM-β-CD in a borate buffer (pH 9.0) although a broad peak was observed due to its degradation. The remaining OPs studied in this work could not be enantiomerically separated. Since malathion is one of the most widely used OPs in agriculture, the developed methodology was applied to its determination in fortified tap water samples using a preconcentration step via an SPE disk to increase the method’s sensitivity. Different elution solvents were evaluated, with ethyl acetate (EtOAc)-diethyl ether (Et_2_O) (50:50) being the one that allowed them to obtain the highest recovery percentage (81 ± 5%). The optimized method made it possible to detect this insecticide in tap water samples, and the authors proposed this method for future studies, such as degradation studies of both enantiomers in environmental samples.

The four stereoisomers of sulfoxaflor, a sulfoximine insecticide with two chiral centers, were separated for the first time by CD-EKC in less than 14 min by Jiménez-Jiménez et al. [19]. It is a potent neurotoxin that causes cell collapse in exposed insects [72,73]. The environmental risk associated with this compound is related to aquatic ecosystems in particular. The separation of the four sulfoxaflor isomers (with resolution values between consecutive peaks of 2.1, 1.5, and 2.6) was achieved with succinyl-β-cyclodextrin (Succ-β-CD) as a chiral selector in a borate buffer at a pH of 9.0. This chiral electrophoretic methodology allowed for the determination of sulfoxaflor enantiomers in commercial agrochemical formulations with an average recovery of 103 ± 3% with respect to the labeled amount.

### 2.2. Herbicides

An herbicide is a chemical product that not only prevents the growth of unwanted weeds, but also reduces fuel consumption as well as tillage [74]. This group of pesticides is one of the most widely used, and, for example, in Spain, it is the second most used type of pesticide per year (constituting approximately 22.6% of the total pesticides employed) [66]. Based on their chemical composition, herbicides can be classified into phenoxy acids, nitrophenols, nitrogen heterocycles, aryl methyl ureas, quaternary salts of heterocycles, halogenated acids and esters, and nitriles [75]. As shown in Table 1, phenoxy acids have been the most analyzed chiral herbicides by CE in water and soil samples, as well as agrochemical formulations. In addition, chiral degradation studies of different herbicides in soil (dichlorprop [34,52], imazaquin [42], metolachlor [41], carfentrazone-ethyl, and carfentrazone [43]) and water (metolachlor [41]) have been carried out.

A sensitive CE methodology was developed by Asami et al., enabling the determination of trace levels of the enantiomers of glufosinate, a phosphorus-containing amino-acid-type herbicide [40]. The use of γ-CD in a phosphate buffer at a pH of 6.5 and fluorescence detection allowed for the separation of glufosinate enantiomers in approximately 6 min with a resolution of 2.5. Given the pK_a_ values of the analyte (pK_a1_ < 2, pK_a2_ = 2.9, pK_a3_ = 9.8) [76], its chiral separation was achieved using a neutral CD thanks to its positive charge due to the protonation of its amino group at the working pH. The method was applied to the enantiomeric determination of glufosinate in fortified river water samples using SPE with titanium oxide as the preconcentration technique and to eliminate matrix components present in the samples, such as inorganic salts and organic compounds. Then, before the CE analysis, large-volume sample stacking (LVSS) was used as the on-line preconcentration technique. For this purpose, a 50-fold diluted solution of the analyte was hydrodynamically injected into the capillary that contained the buffer without CD. Subsequently, a voltage of −30 kV for 10.5–11 min was applied to concentrate the analyte. After this process, the buffer with γ-CD was injected into the capillary at a voltage of +30 kV for 15–20 min, and enantioseparation was achieved. The LOD obtained when using SPE and LVSS was 0.47 µg L^−1^, which showed an important sensitivity improvement with respect to that obtained without the SPE preconcentration (LOD was 35.0 µg L^−1^ when using only LVSS).

Phenoxy acid herbicides are the most stereoselectively studied chemical class of herbicides, most notably dichlorprop (2,4-dichlorophenoxy-2-propionic acid). This compound has a chiral center in its structure, giving rise to two enantiomers. However, only the (+)-isomer has herbicide activity [52], although it is sold and supplied as a racemic mixture. Garrison et al. developed an EKC method allowing for the baseline separation of dichlorprop enantiomers in around 15 min using TM-β-CD as the chiral selector and an acetate buffer at a pH of 4.7 [52]. The chiral methodology was applied to the study of the degradation of dichlorprop enantiomers in soils when a commercial formulation (Foxtril) containing racemic dichlorprop and ioxynil and the nonchiral bifenox ester was used. A UAE with ACN/H_2_O/acetic acid (AcOH) (80:20:2, *v*/*v*/*v*) followed by an LLE with dichloromethane (DCM) and reconstitution of the dry extract with ACN was used for the extraction of the analyte from the spiked soil samples. In addition, a degradation study was carried out in soils from 0 to 31 days after the application of the commercial formulation, and a first-order reaction was observed. The half-life calculated for the degradation of the S-(−)-enantiomer was 4.4 days, while for the R-(+)-enantiomer, it was 8.7 days. At 31 days, none of the enantiomers were present in the soil samples. However, R. Charles concluded in 2004 [77] that all pesticide active ingredients disappear completely in a time equal to five times their half-life. This conclusion was based on experimental data provided by numerous bibliographic references collected in the chapter of Willis and McDowell in 1987 [78]. These considerations do not support the results obtained by Garrison et al. [52] since, based on the study by Charles, the complete disappearance of the S- and R-enantiomers should be at 43.5 days and not at 31 days.

Years later, the stereoselective degradation of the herbicide dichlorprop in soil samples was also investigated by Messina et al. In this case, the enantiomeric separation was based on an optimized method using CEC [34]. A porous homemade monolithic chiral column with a stationary phase of (+)-1-(4-aminobutyl)-(5R,8S,10R)-terguride and a mobile phase of 4 mM triethylamine (TEA)/AcOH in ACN/MeOH (9:1, *v*/*v*) was employed, obtaining the full stereoselective separation of dichlorprop in less than 6 min when using clofibric acid as the internal standard. The extraction of the analyte was carried out by LLE with DCM followed by the evaporation of the solvent and reconstitution of the residue in MeOH. For the study of the stereoselective degradation of dichorprop, the spiked soil sample was separately incubated over 23 days with both the racemate and the enantiomers. The results showed that, when the racemate was incubated, the concentration of both enantiomers decreased, although the S-dichlorprop concentration decreased faster than that of R-dichlorprop. These results confirmed those obtained by Garrison et al. [52]. However, when studying the degradation of the enantiomers separately, the interconversion of the R- to the S-enantiomer and vice versa could be observed. As for the degradation of the R-enantiomer, its initial concentration decreased to 5% after 23 days; however, the S-enantiomer appeared to reach a maximum concentration at 5 days and then also began to degrade. The concentration of the S-enantiomer in the mixture was always lower than that of the R-enantiomer. Regarding the degradation of the S-enantiomer, its concentration decreased to 3.2% after 23 days of incubation. The appearance of the R-enantiomer peaked at 8 days. From this day on, it also began to degrade, although, contrary to the previous case, higher concentrations of the R-enantiomer than the S-enantiomer were present in the mixture, demonstrating an interconversion once again.

**Table 1:** Chiral analysis of pesticides and emerging contaminants by CE in water, soil, agrochemical formulations, and food samples.

Analyte (*Chemical family*)	Applications	Sample Treatment	Separation Conditions	Analysis Time	LOD (Rs)	Ref.
**Pesticides**						
**Insecticides**						
*Cis*-Bifenthrin *(Pyrethroid)*	Enantiomeric analysis of commercial agrochemical formulations.	Dilution of the liquid commercial formulation in MeOH.	BGE: 100 mM borate buffer, pH 8.0 + 20 mM TM-β-CD + 100 mM SC + 2 M Urea Capillary: 50 µm i.d. × 50 cm e.l.; T^a^: 15 °C; V: +30 kV; Injection: 50 mbar × 2 s; Detection: UV 210 nm	9.2 min	4.8 mg L^−1^ (2.8)	[63]
Tetramethrin *(Pyrethroid)*	Enantiomeric analysis of commercial agrochemical formulations.	Dilution of the commercial formulation in the buffer containing 2 M urea and 100 mM SDC.	BGE: 100 mM borate buffer, pH 8.0 + 15 mM HP-β-CD + 50 mM SDC Capillary: 50 µm i.d. × 50 cm e.l.; T^a^: 15 °C; V: +20 kV; Injection: 50 mbar × 2 s; Detection: UV 220 ± 4 nm	<12.5 min	*Trans*-tetramethrin: 1.30 mg L^−1^ (1.7) *Cis*-tetramethrin: 0.97 mg L^−1^ (1.3)	[20]
1- Pyraclofos 2- Profenofos 3- Prothiofos 4- Sulprofos *(Organophosphorus)*	Individual chiral separation. Application in the enantiomeric analysis of soil.	Soil sample was grounded and dried at RT. A volume of 10 mL of MeOH was added to the enriched sample, which was left to stand for 1 h. After shaking for 10 min, the pesticides were extracted with 40 mL MeOH and 10 mg activated charcoal. Then, the mixture was shaken for 30 min, filtered, and extracted with 25 mL MeOH, which was evaporated until it reached 1 mL.	1- BGE: 100 mM SDS + 50 mM SC + MeOH/ACN (4:1, *v*/*v*) 2- BGE: 50 mM SC + 20 mM γ-CD + MeOH/H_2_O/ACN (5:4:1, *v*/*v*/*v*) 3- BGE: 75 mM SC + 20 mM γ-CD + MeOH/H_2_O/ACN (5:4:1, *v*/*v*/*v*) 4- BGE: 50 mM SC + 10 mM γ-CD + MeOH/H_2_O/ACN (5:4:1, *v*/*v*/*v*) Capillary: 50 µm i.d. × 50 cm e.l.; T^a^: 25 °C; V: +30 kV; Injection: 0.5 psi × 5 s; Detection: UV 200 nm	1- 28 min 2- 15 min 3- 22 min 4- 18 min	1- n.d. ^a^ (>2.0) 2- n.d. (1.8) 3- n.d. (1.8) 4- n.d. (1.8)	[36]
1- Malathion 2- Malaoxon 3- Isomalathion 4- Phenthoate 5- Isofenphos 6- Ruelene 7- Phenamiphos 8- Naled *(Organophosphorus)*	Individual chiral separation. Application in the enantiomeric analysis of malathion in tap water.	SPE with ISOLUTE disk (C_8_/ENV +) of the spiked sample. Elution with EtOAc (50:50, *v*/*v*), evaporation of the extract to dry it, and reconstitution of the residue in MeOH.	1, 3, 4, 7- BGE: 25 mM Tris buffer, pH 7.0 + 20 mM CM-β-CD 8- BGE: 25 mM borate buffer, pH 9.0 + 10 mM CM-β-CD Capillary: 75 µm i.d. × 61.5 cm e.l. (1–4) and 50 µm i.d. × 65 cm e.l. (5); T^a^: 25 °C; V: +24 kV; Injection: 50 mbar × 3 s; Detection: UV; 1- and 4- 230 nm; 2- and 3- 254 nm; 5- 214 nm	1- <15 min 2- U ^b^ 3- 17 min 4- 11.8 min 5- U 6- U 7- 12 min 8- <8 min	1- E_1_: 50 mg L^−1^ E_2_: 50 mg L^−1^ (1.4) 3- n.d. (E_1_, E_2_: 2.5 E_3_, E_4_: 1.1) 4- n.d. (2.0) 7- n.d. (0.6) 8- n.d. (>5.0)	[54]
Sulfoxaflor *(Sulfoximine)*	Enantiomeric analysis of commercial agrochemical formulations.	Commercial formulation solutions were diluted in H_2_O, centrifugated, and filtered.	BGE: 100 mM borate buffer, pH 9.0 + 15 mM Succ-β-CD Capillary: 50 µm i.d. × 50 cm e.l.; T^a^: 15 °C; V: +20 kV; Injection: 50 mbar × 8 s; Detection: UV 205 ± 30 nm	13.8 min	E_1_: 0.9 mg L^−1^ E_2_: 1.0 mg L^−1^ (E_1_/E_2_: 2.1) E_3_: 0.9 mg L^−1^; (E_2_/E_3_: 1.5) E_4_: 0.9 mg L^−1^ (E_3_/E_4_: 2.6)	[19]
**Herbicides**						
Glufosinate *(Phosphinate)*	Enantiomeric analysis in river water.	The spiked sample was acidified and mixed with TiO_2_. SPE extraction (elution with NH_3_). Evaporation of the extract to dry it out and reconstitution in Na_2_CO_3_. Prior to CE analysis, in-capillary concentration using LVSS (–30 kV for 10.5–11 min) was determined.	Analyte derivatized with dansyl chloride. BGE: 2 mM phosphate buffer, pH 6.5 + 17 mM γ-CD Capillary: 50 µm i.d. × 69 cm e.l.; T^a^: 25 °C; V: +30 kV; Injection: +30 kV × 17–18 min; Fluorescence detection: λ_excitation_ at 327 nm and λ_emission_ at 557 nm	≈35 min	0.47 µg L^−1^ (2.5)	[40]
Dichlorprop *(Phenoxy acid)*	Enantiomeric analysis of (a) commercial agrochemical formulations; (b) soils. Degradation study in soil.	(a) Commercial formulation was diluted in H_2_O. (b) Extract from spiked soil sample with ACN/H_2_O/AcOH (80:20:2, *v*/*v*/*v*) by UAE. Centrifugation, decantation, and LLE with DCM. Dried out with Na_2_SO_4_, washed with DCM, evaporated to dry it out, and reconstituted in ACN.	BGE: 50 mM acetate buffer, pH 4.7 + 25 mM TM-β-CD Capillary: 75 µm i.d. × 50 cm e.l.; T^a^: 30 °C; V: +20 kV; Injection: hydrodynamic 30 nL × 5 s; Detection: UV 230 nm	16.5 min	(a) 0.1 mg L^−1^ (b) 0.5 mg L^−1^ (2.0)	[52]
Dichlorprop *(Phenoxy acid)*	Enantiomeric analysis and study of stereoselective degradation in soil.	Drying, sieving, spiking of the sample, and followed by incubation in the dark at 20–23 °C for 23 days. Daily extraction of a portion with MeOH, centrifugation, dilution of the supernatant with H_2_O, and adjustment to pH of 2.0. LLE with DCM, evaporation of the organic phase to dry it out, and reconstitution in MeOH.	Mobile phase: 4 mM TEA/AcOH in ACN/MeOH (9:1, *v*/*v*) CEC column: stationary phase of (+)-1-(4-aminobutyl)-(5R,8S,10R)-terguride; 100 µm i.d. × 25.5 cm e.l.; T^a^: 25 °C; V: −15 kV; Injection: −2 kV × 3 s; Detection: UV 254 nm	<6 min	S-dichlorprop 0.46 ng R-dichlorprop 0.42 ng (1.8)	[34]
1- Dichlorprop 2- Fenoprop *(Phenoxy acids)*	Simultaneous enantiomeric analysis in lake water.	SPE with C_18_ membrane disc of the spiked sample, elution with MeOH, and partial evaporation of the extract.	BGE: 100 mM phosphate buffer, pH 5.6 + 1 mM β-CD + 4 mM α-CD Capillary: 50 µm i.d. × 40 cm e.l.; T^a^: 22 °C; V: +25 kV; Injection: pressure × 4 s; Detection: UV 200 nm	<7 min	1- <1 µg L^−1^ (1.2) 2- <1 µg L^−1^ (1.4)	[55]
1- Mecoprop 2- Fenoprop 3- Fluazifop 4- Haloxyfop *(Phenoxy acids)*	Stereoselective simultaneous analysis of acid herbicides in river water and groundwater.	SPE with C_18_H_18_ cartridges and elution with MeOH. L-B-phenyl lactic acid and 37% of NH_3_/MeOH (1:4) were added to the solution and concentrated under vacuum. Solvent was evaporated under a stream of He and redissolved in BR buffer (pH 5.0) containing MeOH (20%, *v*/*v*). WSs were spiked.	BGE: 75 mM BR buffer, pH 5.0 + 10 mM γ-CD + 8 mM VC Capillary: 50 µm i.d. × 33 cm e.l.; Injection: 34.47 kPa × 4 s; T^a^: 25 °C; V: +15 kV; Detection: UV 205 nm	13 min	1 × 10^−6^ M (n.d.)	[37]
1- Fenoprop 2- Mecoprop 3- Dichlorprop 4- 4-CPPA 5- 3-CPPA 6- 2-PPA *(Phenoxy acids)*	Simultaneous enantiomeric analysis in water samples.	WS1 and WS3 were stored for one month and WS2 for three months at 4 °C and then they were filtered. SPE with Oasis HLB and C_18_ cartridges and elution with MeOH. The extract was evaporated to dry it out and reconstituted in 500 µL of MeOH/H_2_O (10:90, *v*/*v*).	BGE: 50 mM phosphate buffer, pH 7.0 + 7 mM HP-β-CD + 20 mM TM-β-CD Capillary: 50 µm i.d. × 50 cm e.l.; T^a^: 15 °C; V: +25 kV; Injection: 50 mbar × 10 s; Detection: UV 4- and 6- 194 nm, 2-, 3-, and 5- 200 nm, and 1- 210 nm	11 min	1- 0.7 mg L^−1^ (1.2) 2- 0.8 mg L^−1^ (2.7) 3- 1 mg L^−1^ (2.0) 4- 1.2 mg L^−1^ (1.7) 5- E_1_: 0.9 E_2_: 0.8 mg L^−1^ (1.2) 6- E_1_: 1.5 E_2_: 1.4 mg L^−1^ (1.6)	[60]
a) 1- 2-phenoxyprop 2- Dichlorprop 3- Fenoprop 4- Fluazifop 5- Haloxyfop 6- Diclofop *(Phenoxy acids)* b) 1- Mecoprop 2- Flamprop 3- Fenoxaprop *(Phenoxy acids)*	Two simultaneous enantiomeric separations of mixtures (a) and (b). Enantiomeric analysis of haloxyfop in soil.	Soil sample was spiked with the commercial herbicide formulation of haloxyfop, followed by hydrolysis. LLE with DCM of the acid hydrolyzate mixed with 1M MeOH/HCl (9:1, *v*/*v*) and H_2_O. Partial evaporation of the organic extract.	BGE: 75 mM BR buffer, pH 5.0 + 6 mM VC Capillary: 50 µm i.d. × 33 cm e.l.; T^a^: 25 °C; V: +20 kV; Injection: 34.5 kPa × 2 s; Detection: UV 210 nm	(a) 8.4 min (b) 8.0 min	(a) 1- n.d. (2.4) 2- n.d. (3.2) 3- n.d. (4.5) 4- n.d. (1.4) 5- 0.19 mg L^−1^ (3.7) 6- n.d. (3.6) (b) 1- n.d. (4.3) 2- n.d. (0.7) 3- n.d. (2.0)	[38]
Metolachlor and its metabolites ESA and OXA *(Chloroacetinalides)*	Enantiomeric analysis and degradation study of metolachlor in water and soil samples.	WS: SPE with C_18_ cartridge of the spiked sample. Elution of metolachlor with EtOAc (analysis by LC-MS) and of OXA and ESA with MeOH (analysis by CE-UV). Evaporation and reconstitution in MeOH/H_2_O (50:50, *v*/*v*). Soil sample: Degradation studies by accelerated extraction with iPrOH SPE of the extract as in the previous section.	BGE: 75 mM borate buffer, pH 9.0 + γ-CD (2.5%, *w*/*v*) + MeOH (20%, *v*/*v*) Capillary: 75 µm i.d. × 50 cm l.e.; T^a^: 15 °C; V: +30 kV; Injection: 0.5 psi × 10 s; Detection: UV	24 min	5 µg L^−1^ (n.d.)	[41]
Imazaquin *(Imidazolinone)*	Enantiomeric analysis and degradation study in soil.	Soil sample was mixed with NaOH, shacked, and centrifuged, and the supernatant was decanted. The extract was acidified (pH 2.8) and centrifuged, and the supernatant was decanted and mixed with DCM by shaking. The DCM extract was centrifuged (to eliminate emulsion and settle any fine particulates). The DCM layers were combined, dried, and then concentrated to near dryness. Then they were redissolved in phosphate buffer (pH 10.1).	BGE: 50 mM phosphate buffer, pH 10.1 + 30 mM HP-β-CD Capillary: 75 µm i.d. × 50 cm e.l.; Injection: 0.5 psi × 8 s; T^a^: 15 °C; +20 kV; Detection: UV 214 nm	14 min	9.7 × 10^−4^–9.8 × 10^−4^ mg kg^−1^ (1.37)	[42]
1- Carfentrazone-ethyl 2- Carfentrazone *(Triazoles)*	(a) Enantiomeric analysis of carfentrazone-ethyl in a commercial herbicide formulation. (b) Enantiomeric analysis and degradation studies of both compounds in sand and soil samples.	(a) Dilution of commercial formulation in MeOH. (b) Spiked sand and soil samples were shaken, incubated for 0, 1, 3, 4, and 7 days, extracted with acetate buffer (pH of 5.0), and centrifuged, and supernatants were collected.	BGE: 25 mM acetate buffer, pH 5.0 + captisol (2.5%, *w*/*v*) Capillary: 50 µm i.d. × 50 cm e.l.; T^a^: 30 °C; V: −30 kV; Injection: 50 mbar × 10 s; Detection: UV 245 ± 4 nm	6.8 min	Carfentrazone-ethyl: E_1_: 0.4 mg L^−1^ E_2_: 0.4 mg L^−1^ (5.1) Carfentrazone: E_1_: 0.3 mg L^−1^ E_2_: 0.3 mg L^−1^ (5.0)	[43]
**Fungicides**						
1- Triadimefon 2- Triadimenol *(Triazoles)*	Simultaneous enantiomeric analysis and soil biotransformation studies of triadimefon in triadimenol.	H_2_O was added to the spiked sample which was incubated at 35 °C for 20 days. Subsequently, LLE with acetone; centrifugation and dilution (1:5, *v*/*v*) of the supernatant with H_2_O; SPE preconcentration with ODS-6 cartridge; elution with acetone; evaporation of the extract to dry it out; and reconstitution in buffer.	BGE:—mM phosphate buffer, pH 3.0 + S-β-CD (2%, *w*/*v*) Capillary: 50 µm i.d. × 53 cm e.l.; T^a^:—°C; V: −20 kV; Injection:—; Detection: UV 220 nm	<30 min	n.d. (n.d.)	[44]
1- Propiconazole 2- Tebuconazole 3- Fenbuconazole *(Triazoles)*	Simultaneous enantiomeric separation and determination in grapes.	Grape samples were chopped and homogenized. Portions of sample were spiked and homogenized with MeOH and H_2_O by sonication. Filtered and passed under vacuum through a C_18_ cartridge. Fungicides were eluted with DCM and concentrated to dry them out. Then, they were reconstituted with buffer solution (without micellar phase).	BGE: 25 mM phosphate buffer, pH 3.0 + 30 mM HP-γ-CD + 50 mM SDS + MeOH/ACN (2:1, *v*/*v*) Capillary: 50 µm i.d. × 56 cm e.l.; Injection: sweeping, 50 mbar × 120 s; T^a^: 20 °C; V: −25 kV; Detection: UV 200 nm	≈17 min	1- 0.1 mg L^−1^ (>1.5) 2- 0.1 mg L^−1^ (>1.5) 3- 0.09 mg L^−1^ (>1.5)	[45]
Propiconazole (1- major enantiomers and 2- minor enantiomers) *(Triazole)*	Enantiomeric analysis and degradation study in two soil–water slurries.	Sample spiking, centrifugation, and filtration.	BGE: 25 mM phosphate buffer, pH 7.0 + 30 mM HP-γ-CD + 75 mM SDS + MeOH (10%, *v*/*v*) + ACN (5%, *v*/*v*) Capillary: 75 µm i.d. × 50 cm e.l.; T: 23 °C; V: +30 kV; Injection: hydrodynamic × 6.5 s; Detection: UV 190 nm	11.9 min	1- 0.75 mg L^−1^ 2- 0.09 mg L^−1^ (2.0)	[78]
1- Prothioconazole 2- Prothioconazole-desthio *(Triazoles)*	(a) Enantiomeric analysis of prothioconazole in commercial agrochemical formulations. (b) Simultaneous enantiomeric analysis of prothioconazole and prothioconazole-desthio and degradation studies in sand and soil samples.	(a) Dilution of the agrochemical formulation in MeOH. (b) Sand and soil samples were spiked with compound racemates, shaken, incubated for 0 and 18 h or 3 and 7 days, extracted with H_2_O, and centrifuged, and the supernatants were collected.	(a) BGE: 100 mM borate buffer, pH 9.0 + 5 mM TM-β-CD Capillary: 50 µm i.d. × 50 cm e.l; T^a^: 15 °C; V: +30 kV; Injection: 50 mbar × 10 s; Detection: UV 205 ± 4 nm (b) BGE: 75 mM borate buffer, pH 9.0 + 10 mM S-γ-CD Capillary: 50 µm i.d. × 50 cm e.l; T^a^: 20 °C; V: +30 kV; Injection: 50 mbar × 6 s; Detection: UV 205 ± 4 nm	(a) 4.5 min (b) 5.5 min	(a) Prothioconazole 0.7 mg L^−1^ (2.8) (b) Prothioconazole 0.9 mg L^−1^ (1.9) Prothioconazole-desthio 1.3 mg L^−1^ (8.2)	[46]
Imazalil *(Imidazol)*	Enantiomeric analysis of imazalil in orange.	Extraction with ACN under basic conditions. The extract was purified by SPE with Sep-Pak plus PS-2 cartridge.	BGE: 50 mM phosphate buffer, pH 3.0 + 4 mM HP-α-CD + 5 mM ammonium dihydrogenphosphate Capillary: 75 µm i.d. × 56 cm e.l.; T^a^: 20 °C; V: +25 kV; Injection: 50 mbar × 2 s; Detection: UV 200 nm	≈14.2 min	0.1 mg L^−1^ (≈6)	[48]
Imazalil *(Imidazol)*	Enantiomeric analysis and study of degradation of racemate in soils.	The samples were spiked, extracted with MeOH, and centrifuged, and the supernatant was partially evaporated and diluted (1:10, *v*/*v*) in buffer.	BGE: 50 mM phosphate buffer, pH 3.0 + 5 mM β-CD Capillary: 75 µm i.d. × 40 cm e.l.; T^a^: 20 °C; V: +25 kV; Injection: 0.5 psi × 5 s; Detection: UV 214 nm	9.5 min	(−)- 0.24 mg L^−1^ (+)- 0.26 mg L^−1^ (4.0)	[47]
Vinclozolin *(Dicarboxamide)*	Enantiomeric analysis in wine samples.	SPE with Sep-Pak plus PS-2 cartridges, elution with ACN and evaporation of the extract. Redissolved in ACN. The extract was injected onto an RSpak DE-613 column with a mobile phase of ACN (62%, *v*/*v*). The fraction containing vinclozolin was combined and diluted with H_2_O. Sample dilution and passed through a Sep-Pak Plus PS-2 cartridge. The resulting residue was redissolved in ACN (20%, *v*/*v*).	BGE: 5 mM borate buffer, pH 8.5 + 50 mM γ-CD + 100 mM SDS + 20 mM phosphate Capillary: 75 µm i.d. × 56 cm e.l.; T^a^: 20 °C; V: +20 kV; Detection: UV 203 nm	≈18.5 min	n.d. (>2)	[56]
1- Metalaxyl 2- Benalaxyl *(Acylamines)*	Individual enantiomeric separation. Application in the chiral analysis in solid and liquid commercial agrochemical samples.	Solid samples: Dissolution in MeOH. Liquid samples: Dilution in MeOH or BGE/H_2_O (50:50, *v*/*v*).	1- BGE: 50 mM MES buffer, pH 6.5 + 15 mM Succ-γ-CD + 2 M Urea 2- BGE: 50 mM MES buffer, pH 6.5 + 5 mM Succ-β-CD + 2 M Urea Capillary: 50 µm i.d. × 50 cm e.l.; T^a^: 15 °C; V: +30 kV, Injection: 25 mbar × 3 s; Detection: UV 210 nm	1- 11.5 min 2- 7.5 min	1- 4.2 mg L^−1^ (3.1) 2- 5.6 mg L^−1^ (15.0)	[64]
**Nematicides**						
1- Fenamiphos and their metabolites (2- fenamiphos sulfone, 3- fenamiphos sulfoxide) *(Organophosphorus)*	Simultaneous enantiomeric analysis in soil.	Drying, crushing, sieving, and spiking of the sample. (1) PLE at 100 °C, 1500 psi for 5 min with EtOH, EtOAc, or heptane (individually or in mixtures) and dried sample with Na_2_SO_4_. (2) SPE: extraction with MeOH and centrifugation. Subsequently, evaporation to dry it out and reconstitution in 5 mM AcOH/NH_3_ buffer (pH of 5.0)-MeOH (15%, *v*/*v*).	BGE: 50 mM ammonium acetate, pH 5.0 + 25 mM CM-β-CD + 10 mM HP-α-CD + MeOH (5%, *v*/*v*) Capillary: 50 µm i.d. × 50 cm e.l.; T^a^: 25 °C; V: +25 kV; Injection: 0.5 psi × 5 s; Detection: UV 214 nm	46 min	1- E_1_: 4.64 mg kg^−1^ E_2_: 4.66 mg kg^−1^ (1.7) 2- E_1_: 0.62 mg kg^−1^ E_2_: 0.51 mg kg^−1^ E_3_: 0.55 mg kg^−1^ E_4_: 0.61 mg kg^−1^ (≈1.5) 3- E_1_: 0.89 mg kg^−1^ E_2_: 0.81 mg kg^−1^ (2.0)	[49]
**Mixtures of different pesticides**						
1- Ruelene *(Organophosphorus insecticide)* 2- Dichlorprop *(Phenoxy acid herbicide)*	Individual enantiomeric analysis and soil degradation studies.	Incubation of the spiked sample with racemates at 25 °C for 6 months.	1- BGE: 20 mM tetraborate buffer, pH 8.5 + 40 mM HP-β-CD + 100 mM SDS + ACN (20%, *v*/*v*) 2- BGE: 25 mM tetraborate buffer, pH 8.5 + 25 mM TM-β-CD Capillary: -; T^a^: -; V: 1- +20 kV and 2- +15 kV; Injection: -; Detection: UV, 1- 200 nm, 2- 230 nm	n.d.	n.d. (n.d.)	[50]
1- Ruelene *(Organophosphorus insecticide)* 2- Dichlorprop *(Phenoxy acid herbicide)* 3- Bromochloroacetic acid *(Haloacetic water disinfectant)*	Individual enantiomeric analyses of 1- and 2- in sludge and 3- in river water.	1, 2- Centrifugation and filtration of the spiked sample with its racemates. Extraction with MeOH, dilution of the extract with H_2_O, centrifugation, and decantation. 3- Filtration and subsequent spiking of the sample with its racemates.	1- BGE 20 mM tetraborate buffer, pH 8.5 + 40 mM HP-β-CD + 100 mM SDS + ACN (20%, *v*/*v*) 2- BGE: 25 mM tetraborate buffer, pH 8.5 + 25 mM TM-β-CD 3- BGE: 50 mM tetraborate buffer, pH 8.5 + 40 mM TM-β-CD Capillary: 75 µm i.d. × 50 cm e.l.; T^a^: 23 °C; V: +25 kV (+15 kV for dichlorprop); Injection: hydrodynamic × 5 s; Detection: UV 1- and 3- 200 nm and 2- 230 nm	1- *Explained in the text* 2- 7.8 min 3- n.d.	1- 5 mg L^−1^ (n.d.) 2- 3 mg L^−1^ (n.d.) 3- 1 mg L^−1^ (n.d.)	[62]
1- Phenothrin *(Pyrethroid insecticide)* 2- Dimethomorph *(Morpholine fungicide)* 3- Bioallethrin *(Pyrethroid insecticide)* 4- Propiconazole *(Triazole fungicide)* 5- Bitertanol *(Triazole fungicide)* 6- Triadimenol *(Triazole fungicide)* 7- Fenpropathrin *(Pyrethroid insecticide)*	Individual enantiomeric analysis in lake water.	Filtration, spiking of the sample with its racemates, and pH adjustment to 3.0. SPE with Oasis HLB cartridge, elution with MTBE/MeOH (90:10, *v*/*v*), evaporation to dry it out, and reconstitution of the residue in MeOH.	1- BGE: 50 mM phosphate buffer, pH 7.0 + 15 mM DM-β-CD + 50 mM SC 2- BGE: 50 mM phosphate buffer, pH 7.0 + 15 mM HP-γ-CD + 50 mM SC 3- BGE: 50 mM phosphate buffer, pH 7.0 + 15 mM HP-β-CD + 50 mM SC 4- BGE: 50 mM phosphate buffer, pH 7.0 + 15 mM TM-β-CD + 50 mM SDS 5- BGE: 50 mM phosphate buffer, pH 7.0 + 15 mM TM-β-CD + 50 mM SDS 6- BGE: 50 mM phosphate buffer, pH 7.0 + 15 mM HP-γ-CD + 50 mM SDS 7- BGE: 50 mM phosphate buffer, pH 7.0 + 15 mM γ-CD + 50 mM SDS Capillary: 50 µm i.d. × 40 cm e.l.; T^a^: 20 °C; V: +20 kV; Injection: 3.5 kPa × 2 s; Detection: UV 214 nm	1- 6 min 2- 8.1 min 3- 8.5 min 4- 11.5 min 5- 11.8 min 6- 12 min 7- 17.8 min	1- 0.98 µg L^−1^ (1.5) 2- 0.18 µg L^−1^ (8.7) 3- 0.41 µg L^−1^ (2.4) 4- 0.27 µg L^−1^ (1.5) 5- 0.40 µg L^−1^ (1.5) 6- 0.60 µg L^−1^ (1.5) 7- 0.36 µg L^−1^ (7.1)	[57]
1- λ-Cyhalothrin *(Pyrethroid insecticide)* 2- β-Cyfluthrin *(Pyrethroid insecticide)* 3- *Cis*-bifenthrin *(Pyrethroid insecticide)* 4- Resmethrin *(Pyrethroid insecticide)* 5- Diniconazole *(Triazole fungicide)* 6- Metalaxyl *(Acylamine fungicide)* 7- Benalaxyl *(Acylamine fungicide)* 8- Hexaconazole *(Triazole fungicide)* 9- Myclobutanil *(Triazole fungicide)* 10- Tebuconazole *(Triazole fungicide)* 11- Dichlorprop *(Aryloxy propionic acids)* 12- Mecoprop *(Aryloxy propionic acids)* 13- α-Cypermethrin *(Pyrethroid insecticide)* 14- Uniconazole *(Triazole fungicide)* 15- Flutriafol *(Triazole fungicide)* 16- Fenpropathrin *(Pyrethroid insecticide)*	Individual enantiomeric separations. Enantiomeric analysis of metalaxyl and its enantiomeric impurity in a commercial fungicide product marketed as enantiomerically pure (metalaxyl-M) and in soil and tap water samples.	Dried soil sample was spiked with pure commercial product (R-metalaxyl). Extraction with MeOH, partial evaporation, dilution with H_2_O, and LLE with EtOAc. Drying of the organic phase with Na_2_SO_4_ and reconstitution in hexane. Finally, SPE was cleaned with Si cartridge, followed by elution with EtOAc/hexane (20:80, *v*/*v*), evaporation to dry it out, and reconstitution in ACN/H_2_O (80:20, *v*/*v*). Spiked tap WS with pure commercial product (R-metalaxyl) was extracted with C_18_ cartridge, eluted with MeOH, evaporated to dry it out, and reconstituted in ACN/H_2_O (80:20, *v*/*v*).	Mobile phase: ACN/H_2_O/ammonium formate (90:9:1, *v*/*v*/*v*) pH 2.5 CEC column: stationary phase of tris cellulose (4-chloro-3-methylphenylcarbamate); 100 µm i.d. × 24 cm e.l.; T^a^: 25 °C; V: −10 kV; Injection: 10 bar × 12 s; Detection: UV 210 nm	1- U 2- U 3- U 4- U 5- 18.1 min 6- 14.7 min 7- 19.2 min 8- 24.5 min 9- 20.3 min 10- 21.5 min 11- U 12- U 13- U 14- 21.9 min 15- 18.5 min 16- U	5- n.d. (2.7) 6- 1.4 (S-metalaxyl; impurity) and 1.6 (R-metalaxyl) mg L^−1^ (2.5) 7- n.d. (1.3) 8- n.d. (6.4) 9- n.d. (0.8) 10- n.d. (2.6) 14- n.d. (3.3) 15- n.d. (0.9)	[35]
**Drugs**						
**Antihypertensive**						
1- Pindolol 2- Atenolol 3- Propranolol 4- Metoprolol *(Hydroxypropyl amines)*	Simultaneous enantiomeric analyses in river, tap, and groundwater.	SPE with SBA_15_-C_18_ extraction cartridge of the spiked samples with racemates. Elution with MeOH, evaporation of the extract to dry it out, and reconstitution of the residue in BGE.	BGE: 50 mM phosphate buffer, pH 2.5 + M-β-CD (1.25%, *w*/*v*) Capillary: 50 µm i.d. × 40 cm e.l.; T^a^: 30 °C; V: +20 kV; Injection: +10 kV × 6 s; Detection: UV, 1- and 3- 220 nm, 2- and 4- 200 nm	<35 min	1- 1.3 µg L^−1^ (1- 1.5) 2- 1.3 µg L^−1^ (2- 1.1) 3- S-1.3 µg L^−1^ R-1 µg L^−1^ (3- 2.9) 4- 1.6 µg L^−1^ (4- 1.3)	[58]
1- Pindolol 2- Atenolol 3- Propranolol 4- Metoprolol *(Hydroxypropyl amines)*	Simultaneous enantiomeric analyses in river and sewage water samples.	SPE with SBA_15_-C_8_ extraction cartridge of the spiked samples with racemates. Elution with MeOH, evaporation of the extract to dry it out, and reconstitution of the residue in BGE.	BGE: 50 mM phosphate buffer, pH 2.5 + M-β-CD (1.25%, *w*/*v*) Capillary: 50 µm i.d. × 41 cm e.l.; T^a^: 20 °C; V: +20 kV; Injection: 5 kPa × 5 s; Detection: UV, 1- and 3- 220 nm, 2- and 4- 200 nm	<42 min	1- 0.5 µg L^−1^ (1- n.d.) 2- 0.5 µg L^−1^ (2- n.d.) 3- 0.4 µg L^−1^ (3- n.d.) 4- 0.6 µg L^−1^ (4- n.d.)	[59]
**Anti-inflammatory**						
Ketoprofen *(2-aryl propionate)*	Enantiomeric analysis in wastewater.	The sample was stabilized with nitric acid (0.1%, *v*/*v*) and stored in dark at 8 °C. Samples were spiked with the compounds. An on-line preconcentration step was necessary, using 50 mmol L^−1^ borate/NaOH electrolyte at pH of 9.5 containing MeOH 0–80% (*v*/*v*).	BGE: 50 mM phosphate buffer, pH 2.5 + S-β-CD (4%, *w*/*v*) + TM-β-CD (0.5%, *w*/*v*) + 20 mM SDS Capillary: 50 µm i.d. × 24.5 cm e.l.; T^a^: 25 °C; V: −15 kV; Injection: −15 kV × 30 min; Detection: UV 200 nm	13.5 min	E_1_: 0.64 µg L^−1^ E_2_: 0.86 µg L^−1^ (>2.0)	[61]
Ibuprofen *(2-aryl propionate)*	Enantiomeric analyses in urban water and human urine samples.	DSPE for sample solution with pH of 4.0 containing 1.4 M NaCl and 0.12 g adsorbent (MoS_2_). 10 min adsorption time at 25 °C for 1 mL the elution solvent (acetone-0.25 M NaOH (aq) (2:1, *v*/*v*)), and 5 min desorption time at 50 °C. A rate of 900 rpm was used for adsorption and desorption steps.	BGE: 100 mM phosphate buffer, pH 6.5 + 1 mM VC Capillary: 50 µm i.d. × 47 cm e.l.; T^a^: 20 °C; V: +20 kV; Injection: 65 mbar × 10 s; Detection: UV 214 nm	26 min	0.025 mg L^−1^ (n.d.)	[39]
**Mixtures of different drug families**						
1- Duloxetine *(Amine)* 2- Terbutaline *(Hydroxypropyl amine)* 3- Econazole *(Imidazole)* 4- Propranolol *(Hydroxypropyl amine)* 5- Verapamil *(Nitrilo)* 6- Metoprolol *(Hydroxypropyl amine)* 7- Betaxolol *(Hydroxypropyl amine)*	Simultaneous enantiomeric analysis in wastewater.	Samples were filtered and stored at 4 °C before being analyzed.	BGE: 25 mM phosphate buffer, pH 3.0 + S-β-CD (2%, *w*/*v*) Capillary: 50 µm i.d. × 50 cm e.l.; T^a^: 20 °C; V: −20 kV; Injection: 50 mbar × 10 s; Detection: UV, 1- 220 nm, 2-, 3-and 5- 200 nm, 4- 215 nm, 6- and 7- 194 nm	16 min	1- 0.5 mg L^−1^ (8.3) 2- 0.7 mg L^−1^ (8.4) 3- 1.5 mg L^−1^ (8.5) 4- 0.4 mg L^−1^ (4.1) 5- 0.6 mg L^−1^ (3.7) 6- 0.7 mg L^−1^ (2.5) 7- 0.8 mg L^−1^ (2.4)	[53]

^a^ n.d.: no data in the reference article. ^b^ U: unseparated enantiomerically. ACN: acetonitrile; aq: aqueous; BGE: background electrolyte; BR: Britton–Robinson buffer (40 mM boric acid/40 mM phosphoric acid/40 mM acetic acid); 2-PPA: 2-phenoxy propionic acid; 3-CPPA: 2-(3-chlorophenoxy) propionic acid; 4-CPPA: 2-(4-chlorophenoxy) propionic acid; C_18_H_18_: styrene-divinylbenzene; CEC: capillary electrochromatography; CM-β-CD: carboxymethyl-β-cyclodextrin; DCM: dichloromethane; DM-β-CD: heptakis (2,6-di-O-methyl)-β-cyclodextrin; DSPE: dispersive solid-phase extraction; E_1_: first-migration enantiomer; E_2_: second-migration enantiomer; E_3_: third-migration enantiomer; E_4_: fourth-migration enantiomer; e.l.: effective length; ESA: ethane sulfonic acid; Et_2_O: diethyl ether; EtOAc: ethyl acetate; EtOH: ethanol; H_2_O: water; HCl: hydrochloride; He: helium; HP-β-CD: 2-hydroxypropyl-β-cyclodextrin; HP-γ-CD: 2-hydroxypropyl-γ-cyclodextrin; AcOH: acetic acid; HP-α-CD: 2-hydroxypropyl-α-cyclodextrin; i.d.: internal diameter; ISOLUTE (C_8_/ENV +): polymeric and hydroxylated extraction sorbent functionalized with C_8_ chains; iPrOH: isopropanol; LLE: liquid–liquid extraction; LOD: limit of detection; LVSS: large-volume sample stacking; M-β-CD: methylated-β-cyclodextrin; MeOH: methanol; MES: 2-morpholinoethanesulfonic acid; MONTH: n-(N-morpholino) ethane sulfonic acid; MoS_2_: molybdenum disulphide; MS: mass spectrometry; MTBE: methyl tert-butyl ether; Na_2_CO_3_: sodium carbonate; Na_2_SO_4_: sodium sulfate; NH_3_: ammonia; ODS-6: silica extraction sorbent functionalized with C_18_ chains; OXA: oxanilic acid; PLE: pressurized liquid Extraction; Rs: electrophoretic resolution of the enantiomers; RT: room temperature; S-β-CD: sulphated-β-CD; S-γ-CD: sulphated-γ-CD; SBA_15_-C_18_: functional mesoporous extraction sorbent with C_18_ chains; SC: sodium cholate; SDS: sodium dodecyl sulphate; Sep-Pak plus PS-2: styrene-divinylbenzene copolymer extraction sorbent; SPE: solid-phase extraction; Succ-β-CD: succinyl-β-cyclodextrin; Succ-γ-CD: succinyl-γ-cyclodextrin; TEA: triethylamine; TFA: trifluoroacetic acid; TiO_2_: titanium oxide; TM-β-CD: heptakis (2,3,6-tri-O-methyl)-β-cyclodextrin; UAE: ultrasound-assisted extraction; UV: ultraviolet; VC: vancomycin; WS: water sample.

The simultaneous enantiomeric analysis of different phenoxy acid herbicides by EKC using mixtures of chiral selectors (neutral CDs (β-CD, α-CD, γ-CD, HP-β-CD, TM-β-CD) and/or VC) was performed in spiked water samples [37,55,60]. Hsieh et al. studied the simultaneous separation of seven chlorophenoxy acids (2,4-dichlorophenoxyacetic acid, dichlorprop, 4-(2,4-dichlorophenoxy) butyric acid, 4-chloro-2-methylphenoxyacetic acid, 4-(4-chloro-2-methylphenoxy) butyric acid, 2,4,5-trichlorophenoxyacetic acid, and 2-(2,4,5-trichlorophenoxy) propionic acid (known as fenoprop)), from which two had a chiral center (dichlorprop and fenoprop) using CDs in the separation buffer [55]. It was shown that the cavity size and the concentration of CDs greatly influenced the analysis time needed for the simultaneous separation. The use of a mixture of two neutral CDs (β-CD and α-CD) in a phosphate buffer at a pH of 5.6 allowed for the simultaneous separation of all compounds, including the complete enantioseparation of the pairs of enantiomers of dichlorprop and fenoprop in less than 7 min. The method was applied to the determination of the seven compounds in spiked lake water after their preconcentration by SPE with C_18_ discs and elution with MeOH. The detection limits reached were lower than 1 µg L^−1^.

**Figure 1:**
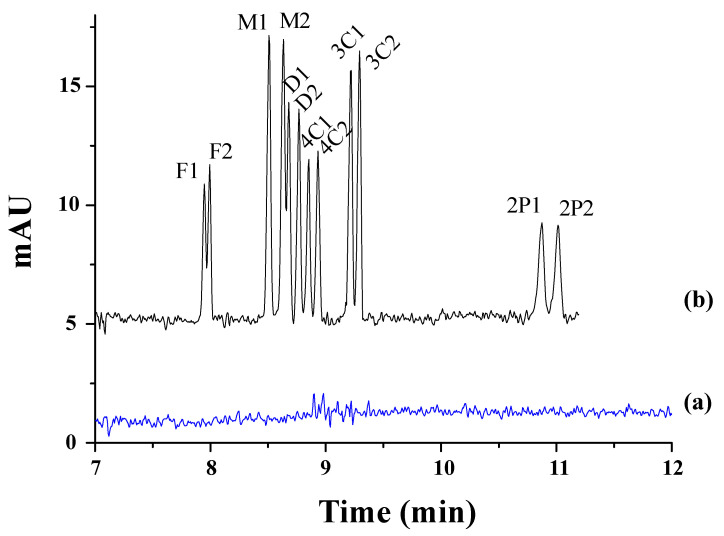
Electropherograms obtained for (a) non-spiked water sample and (b) spiked water sample with the mixture of six phenoxy acid herbicides using 100 mg of PMO-STPA sorbent and a 750 mL sample volume. Spiked concentrations of 3.3 µg L^−1^ for the six phenoxy acids. Compounds: F: fenoprop (Rs = 1.1), M: mecoprop (Rs = 2.7), D: dichlorprop (Rs = 1.9), 4C: 4-CPPA (Rs = 1.7), 3C: 3-CPPA (Rs = 1.2), 2P: 2-PPA (Rs = 1.6), 1: First-migration enantiomer; 2: Second-migration enantiomer. Experimental conditions: BGE: 50 mM phosphate buffer (pH 7.0) + 20 mM TM-β-CD + 7 HP-β-CD, uncoated fused-silica capillary effective length of 50 µm i.d. *×* 50 cm, injection by pressure of 50 mbar *×* 10 s, temperature of 15 °C, applied voltage of +25 kV, and UV detection at 194 nm (2-PPA and 4-CPPA), 200 nm (M, D, and 3-CPPA), and 210 nm (F) (bandwidth 5 nm). Reproduced with permission [60].

Polcaro et al. reported a method allowing for simultaneous enantiomeric analyses of four phenoxy acid herbicides (mecoprop, fenoprop, fluaziprop, and haloxyfop) in ground and river water samples [37]. They used a dual system based on γ-CD combined with VC and a Briton–Robinson buffer at a pH of 5.0. However, the sensitivity was very limited with detection limits of 10^−6^ M, and therefore, the analytes were extracted from the water samples by SPE with styrene-divinylbenzene cartridges, which improved the detection sensitivity to 4 × 10^−10^–2 × 10^−9^ M and minimized sample contamination. Valimaña-Traverso et al. described the simultaneous enantiomeric separation of six phenoxy acid herbicides (fenoprop, mecoprop, dichlorprop, 2-(4-chlorophenoxy) propionic acid (4-CPPA), 2-(3-chlorophenoxy) propionic acid (3-CPPA), and 2-phenoxypropionic acid (2-PPA)) using a dual CD system (TM-β-CD and HP-β-CD) and a phosphate buffer at a pH of 7.0 [60]. The chiral separation took place in 11 min with resolution values from 1.2 to 2.7. Two novel periodic mesoporous organosilica materials (styrylmethyl)bis(triethoxysilylpropyl)ammonium chloride (PMO-STPA) and bis(3-triethoxysilylpropyl)amine (PMO-TEPA)) were evaluated as sorbents in SPE for the preconcentration of two mixtures of six phenoxy acid herbicides in water samples prior to their analysis by CE. The best recoveries were obtained with the PMO-STPA sorbent. The analysis of river samples and effluents from wastewater treatment plants using the developed CE method gave rise to recoveries ranging from 78.3 to 107.5% and LODs from 0.7 to 1.5 mg L^−1^. Figure 1 shows, as an example, the electropherograms corresponding to the separation of the enantiomers of the mixture of the six phenoxy acid herbicides studied in this work in spiked and non-spiked water samples with the PMO-STPA sorbent (100 mg) and a 750 mL sample volume under the optimized conditions.

Desiderio et al. used the macrocyclic antibiotic VC as the sole chiral selector in CE to achieve the enantioseparation of mecoprop, fenoprop, dichlorprop, haloxyfop, and fluazifop, as well as the enantioseparation of four chiral phenoxy acid herbicides [38]. On the one hand, the simultaneous enantiomeric separation of 2-phenoxyprop, dichlorprop, fenoprop, fluazifop, haloxyfop, and diclofop was carried out in less than 8 min, while, on the other hand, the enantiomers of the other compounds were simultaneously separated in less than 8.4 min. In addition, the authors achieved the best resolution values for the analytes that had been previously enantioseparated. A partial filling method was used to avoid VC reaching the detection path since this antibiotic strongly absorbs UV radiation, causing a loss in sensitivity. Different variables, such as the pH of the BGE, temperature, and VC concentration, were shown to influence the enantioresolution values and the selectivity of the separation. Using a 6 mM concentration of VC, a temperature of 25 °C, and a Britton–Robinson buffer at a pH of 5.0, baseline resolutions were obtained for all the studied compounds. The optimized methodology was applied for the determination of haloxyfop in soil samples spiked with a racemic mixture of a commercial herbicide formulation. The proposed partial filling method with VC was advantageous in that it was fast and inexpensive with respect to other techniques that require expensive chiral stationary phases or derivatization protocols.

Klein et al. separated the enantiomers of metolachlor by liquid chromatography (LC) and achieved the simultaneous separation of the enantiomers of its two polar metabolites, ethane sulfonic acid (ESA) and oxanilic acid (OXA) by EKC [41]. The three compounds contain a chiral axis and a chiral center, so four peaks were observed for each one in the electropherograms. Metolachlor was enantioseparated employing a cellulose tris(3,5-dimethylphenyl carbamate) chiral stationary phase. However, for both metabolites, γ-CD as the chiral selector in the presence of MeOH at a pH of 9.0 was used. The chiral separation of metolachlor was achieved in a shorter time (less than 12 min) than that of the electrophoretic separation of both metabolites (less than 25 min). Both the LC and EKC methodologies were applied for the enantiomeric determination of metolachlor and its metabolites, respectively, in water and soil samples, as well as for degradation studies in these samples. The results showed that metolachlor degradation was not enantioselective and that racemization did not take place under these conditions. The method developed by EKC can be applied for the analysis of samples with high concentrations of ESA and OXA, if a previous SPE procedure is carried out to avoid all possible interferences.

Imazaquin is an imidazolinone herbicide with three different ionization pk_a_ values (1.8, 3.8, and 10.5). A simple CE method using HP-β-CD at a pH of 10.1, in which imazaquin is negatively charged, allowed for the baseline enantioseparation of this compound with an analysis time of 14 min [42]. The optimized method was applied to the study of enantioselective degradation in field soil over 21 days, observing that the enantiomers’ degradation was slightly different (the half-life of one enantiomer was 9.3 and for the other it was 8.3 days) and the soil pH strongly influenced the enantiomeric degradation.

**Figure 2:**
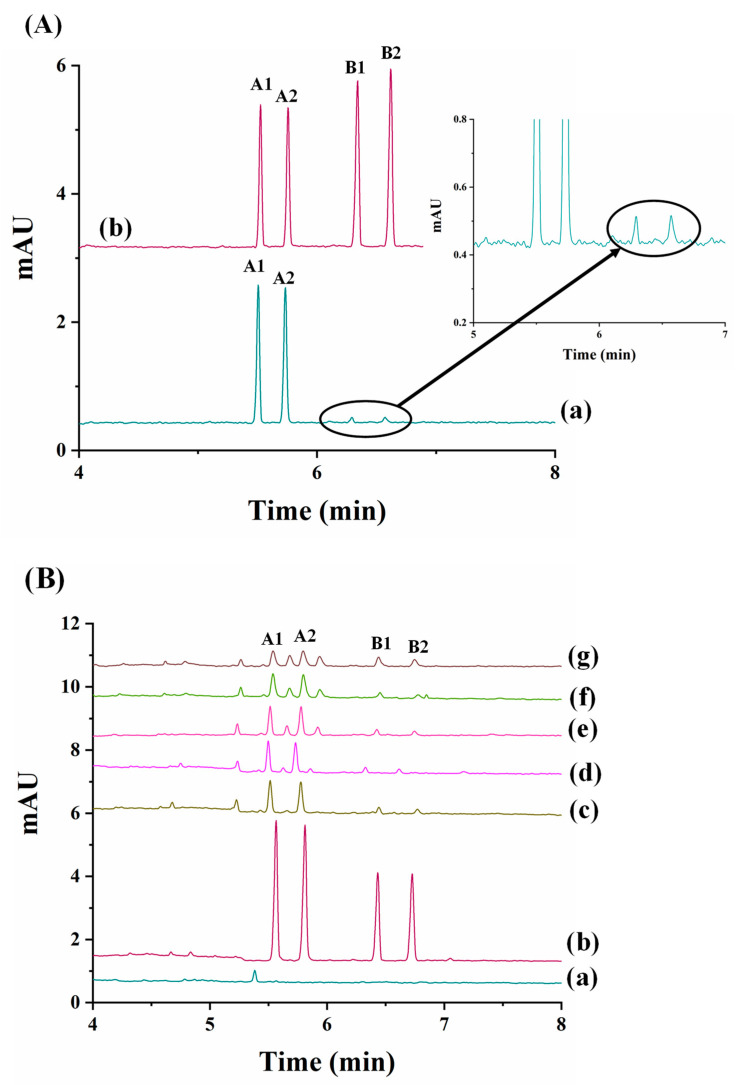
Electropherograms corresponding to: (**A**) The analysis of a carfentrazone-ethyl-based commercial agrochemical formulation: (a) standard solution containing 30 mg L^−1^ of carfentrazone-ethyl racemate and 20 mg L^−1^ of carfentrazone racemate, and (b) commercial herbicide formulation containing 30 mg L^−1^ of carfentrazone-ethyl racemate according to the label. (**B**) The analysis of the extracts obtained from soil samples spiked with 40 mg L^−1^ carfentrazone-ethyl racemate (degradation study of carfentrazone-ethyl in soil samples): (a) soil extract blank; (b) standard solution of carfentrazone-ethyl racemate and carfentrazone racemate at 40 mg L^−1^ and 20 mg L^−1^, respectively; (c) soil extract after zero days; (d) soil extract after one day; (e) soil extract after three days; (f) soil extract after four days; and (g) soil extract after seven days. Experimental conditions: BGE: 25 mM sodium acetate buffer (pH of 5.0) + 2.5% (*w*/*v*) captisol, uncoated fused-silica capillary with an effective length of 50 µm i.d. *×* 50 cm, injection by pressure of 50 mbar *×* 10 s, temperature of 30 °C, applied voltage of −30 kV, and UV detection at 245 ± 4 nm. A1 and A2: carfentrazone-ethyl enantiomers; B1 and B2: carfentrazone enantiomers. Reproduced with permission [43].

The most recent work dealing with the enantioseparation of herbicides was carried out by García-Cansino et al. [43]. The simultaneous enantiomeric separation of carfentrazone-ethyl, a chiral postemergence herbicide, and its acid metabolite (carfentrazone) was achieved with an analysis time of 6.8 min with enantiomeric resolutions of 5.0 and 5.1, respectively. An anionic CD (captisol) was used as chiral selector at a concentration of 2.5% *w*/*v* at a pH of 3.0. This methodology was applied for the determination of carfentrazone-ethyl in an agrochemical formulation, and the peaks of the carfentrazone-ethyl enantiomers and two additional peaks of the carfentrazone enantiomers were observed (see Figure 2A).

Moreover, the degradation of both compounds in clean sand and soil samples was investigated. The results did not show a significant level of degradation for any of the compounds in clean sand samples. Regarding soil samples, the level of carfentrazone degradation could reach 15% on the seventh day while a significant but not stereoselective level of degradation was observed for carfentrazone-ethyl (80%), with up to a 9.5% enrichment observed for carfentrazone (see Figure 2B). The results demonstrated the potential of the method to control the quality of agrochemical formulations and to investigate the stereoselectivity of degradation processes in environmental samples.

### 2.3. Fungicides

Fungicides are used to avoid or prevent the growth of fungi and molds harmful to plants or animals. They are the most frequently employed pesticides. A clear example is observed in Spain, where approximately half of the pesticides employed annually are fungicides (45.2%) [66]. From the long list of fungicide families, conazole and amide types have been the most frequently studied in terms of the impact of chirality on their properties [64]. To our knowledge, only eight articles have been devoted to the chiral separation of these compounds by CE (Table 1), with specifically triazole, imidazole, dicarboxamide, and acylamine being the fungicide groups separated. Chiral methods have been applied for the determination and degradation studies of the enantiomers of these compounds in soil samples [44,46,47,79], for the analysis of agrochemical formulations [46,64], and for their determination in food samples such as oranges, wine, and grapes [45,48,56], being the only group of pesticides that have been analyzed in food samples.

Triadimefon is the most important triazole fungicide since its introduction in the market in 1970, with an important antifungal activity due to its transformation to triadimenol, also used as a fungicide [44]. Considering their chemical structures, while triadimefon has a single chiral center generating a pair of enantiomers, triadimenol contains two chiral centers, generating four stereoisomers. The simultaneous separation of both enantiomers of triadimefon and the four stereoisomers of triadimenol was achieved with sulphated-β-cyclodextrin (S-β-CD) at a pH of 3.0 and in the negative polarity mode [44]. A good enantioselectivity was obtained for both compounds under optimal experimental conditions but with a long analysis time (≈30 min). The application of the method to a stereoselective study associated with the biotransformation of triadimefon into triadimenol by soil microorganisms was illustrated. The methodology was compared to the commonly employed chiral GC method, which revealed that, using CE, better results were obtained in terms of selectivity and sensitivity in the analysis of real samples.

The chiral determination of three other triazole fungicides, propiconazole (two chiral centers), fenbuconazole, and tebuconazole, in spiked grape samples was carried out by CD-MEKC using HP-γ-CD as the chiral selector and SDS as the surfactant. This was the first time that the four enantiomers of propiconazole were separated by CE [45]. Two phosphate buffers, one at a pH of 7.0 and the other at a pH of 3.0 containing 10% MeOH and 5% ACN were used. The effect of the type of on-line sample preconcentration techniques (normal injection, stacking injection, or sweeping injection) was investigated to obtain the best sensitivity in an efficient and versatile way. The sweeping-CD-MEKC method under acidic conditions gave rise to the best detection sensitivity with LODs for the enantiomers of the studied triazoles close to 0.1 mg L^−1^. Combining this methodology with an extraction of the analytes by SPE using C_18_ cartridges and dichloromethane as the eluent resulted in recoveries from spiked grapes samples ranging from 73 to 109%. Garrison et al. developed another CD-MEKC methodology for the separation of the four stereoisomers of propiconazole that was very similar to the previous work [79]. Their method enabled them to follow the loss of propiconazole stereoisomers from the water phase of two different soil–water slurries under aerobic conditions. GC mass spectrometry (MS) with a chiral column was also employed as a comparative technique. The described methodology implied the use of HP-γ-CD as the chiral selector, as in the previous work [45], and SDS as the surfactant, but, in this case, a phosphate buffer at a pH of 7.0 in the presence of 10% MeOH and 5% of ACN allowed them to slightly improve their resolution values and decrease the analysis time in 5 min. Then, a degradation study in soil–water slurries under aerobic conditions was carried out over five months, and the half-time was observed to be 45 and 51 days for the two slurries studied. The authors concluded that the four propiconazole stereoisomers showed an equivalent loss from the aqueous phase of the slurries and therefore little or no stereoselectivity. Prothioconazole is another chiral triazole fungicide widely employed for the treatment of crops such as soybean and cereals. Prothioconazole can be degraded in animals, plants, and soils by desulfurization to prothioconazole-desthio (its main metabolite), which is also chiral and presents a greater level of mammalian toxicity than prothioconazole [46]. The fast and cost-effective enantiomeric separation of prothioconazole and its determination in agrochemical formulations were achieved by CE in 4.5 min with a resolution of 2.8 [46] using TM-β-CD as the chiral selector in borate buffer at a pH of 9.0. Moreover, due to the neutral nature of prothioconazole-desthio, an anionic CD (sulphated-γ-cyclodextrin (S-γ-CD)) was selected to achieve the simultaneous enantiomeric separation of prothioconazole and prothioconazole-desthio in 5.5 min with resolutions of 1.9 and 8.2, respectively [46]. The evaluation of the analytical characteristics of the two CE methods showed their performance to quantify prothioconazole in commercial agrochemical formulations and to investigate the degradation of both compounds in soil and sand samples. A higher degradation was observed for prothioconazole in soils (≈50%) than in sand samples (≈40%), while prothioconazole-desthio showed similar levels of degradation for both matrices (40%). No enantioselective degradation was observed for any of the compounds studied in any of the samples (sand and soil).

**Figure 3:**
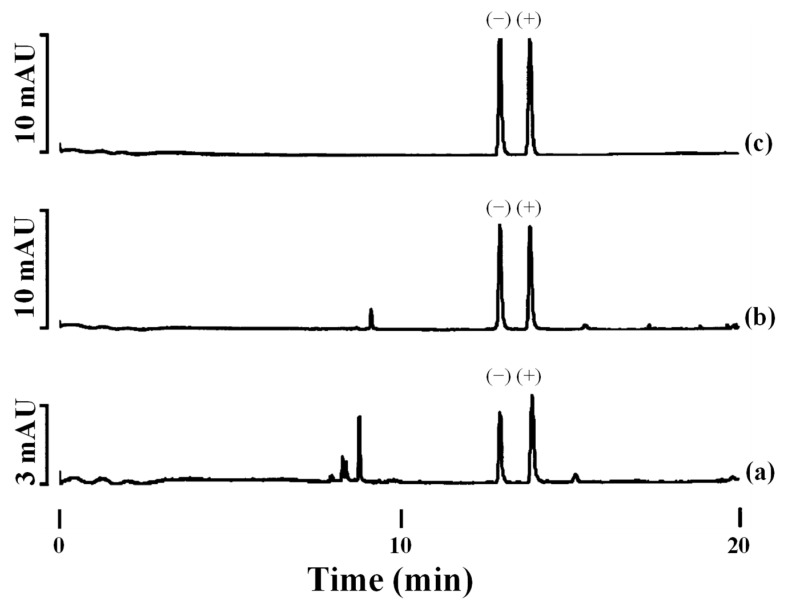
Electropherograms of imazalil enantiomers in: (**a**) orange sample (2); (**b**) orange sample (1); and (**c**) standard solution (20 mg L^−1^ racemic imazalil). (−) and (+) represent (−)- and (+)-imazalil; Experimental conditions: BGE: 5 mM ammonium dihydrogenophosphate—50 mM phosphate buffer (pH 3.0) + 4 mM 2HP-β-CD, 75 µm id × 56 cm e.l. uncoated fused-silica capillary, injection by pressure of 50 mbar *×* 2s, temperature of 20 °C, applied voltage of +25 kV, and UV detection at 200 nm. Reproduced with permission [48].

In contrast to the three nitrogen atoms in the azole ring of triazoles, imidazole fungicides possess two nitrogen atoms. Imazalil is a systemic imidazole fungicide used to control fungal diseases in fruits and vegetables by inhibiting ergosterol biosynthesis [47,48]. Two articles have reported the development of chiral methods for the separation of imazalil using CE with CDs as chiral selectors [47,48]. First, 2-hydroxypropyl-α-cyclodextrin (HP-α-CD) was used as the chiral selector at a pH of 3.0 for the separation of imazalil enantiomers and to determine the enantioselectivity of (+)- and (−)-imazalil residues in oranges [48]. Under optimal experimental conditions, a resolution value of 6.0 and an analysis time approximately of 14.2 min were obtained. A mixture of can (30 mL) with 1 M sodium hydroxide (NaOH) (1 mL) was used for imazail’s extraction from the orange samples followed by extract purification by SPE. From the eight orange samples analyzed, imazalil was found in seven of them. Figure 3 depicts the electropherograms obtained for two orange samples (Figure 3a,b) and for a standard 20 mg L^−1^ solution in racemic imazalil (Figure 3c). The total levels of (−)- and (+)-imazalil in these seven samples ranged from 0.64 to 1.95 mg kg^−1^. In four of them, the total levels of both enantiomers were the same (one of them, orange sample 1, is shown in Figure 3b); however, in the remaining three, the total levels were different between enantiomers: 45:55, 48:52, and 42:58 (this last, orange sample 2, is shown in Figure 3a). As in the European Union and Japan the maximum allowed concentration of imazalil is 5 mg kg^−1^ for citrus fruits [80], this work illustrated that the values found in the oranges were below the maximum permitted limits [48].

In the second work, Chu et al. demonstrated the degradation of the two enantiomers of imazalil in soil samples [47]. With this purpose, a method based on the use of β-CD at a pH of 3.0 was used. Analysis times less than 10 min and LODs of 0.24 and 0.26 µg mL^−1^ were obtained for (−)-imazalil and (+)-imazalil, respectively. Five studies were carried out under different soil conditions, obtaining the following, in decreasing order of degradation: UV irradiation > sunlight > soil with planted wheat > sterilized soil > soil kept in the dark.

Kodama et al. reported the stereoselective separation by CD-MEKC of the fungicide vinclozolin, a dicarboximide fungicide with androgenic activity used in Europe to protect fruits, vegetables, ornamental plants, and turf grasses [56]. Under optimized experimental conditions, using γ-CD as the chiral selector combined with SDS at a pH of 8.5, the enantioseparation of vinclozolin was achieved with an analysis time of approximately 19 min, reaching a resolution of 2.1. The determination of vinclozolin enantiomers in wine samples prior to solvent extraction by SPE with ACN (62%, *v*/*v*) was carried out. It was observed that the peak areas of the (+)- and (−)-enantiomers in wine had the ratio of 2:3, i.e., they were not racemic. Thus, it was shown that the (+)-enantiomer degraded more rapidly than the (−)-enantiomer. In addition, the antiandrogenic activity of each vinclozolin enantiomer was studied, and it was stronger for (+)-vinclozolin.

Pérez-Fernández et al. developed a CD-EKC methodology enabling the individual enantiomeric separation of two acylamine fungicides, metalaxyl and benalaxyl [64]. The separation method was based on the use of 2-morpholinoethanesulfonic acid (MES) buffer and urea as the separation medium, employing succinyl-γ-cyclodextrin (Succ-γ-CD) and Succ-β-CD as chiral selectors for metalaxyl and benalaxyl, respectively. These methods were applied for the determination of each fungicide in solid and liquid commercial agrochemical formulations which were dissolved in MeOH or diluted in MeOH or BGE/H_2_O (50:50, *v*/*v*), respectively. A resolution value of 3.1 was obtained for the separation of the metalaxyl enantiomers in 11.5 min, while for benalaxyl, a resolution close to 15 was reached with an analysis time of 7.5 min. The proposed CE methods made the enantioseparation of benalaxyl possible for the first time and also showed a clear improvement in the analysis time and resolution for the chiral separation of metalaxyl, compared with those of the previously published article by CE [51], which will be discussed in Section 2.5. Also, a stacking on-line preconcentration strategy was implemented to improve the LODs of the method for metalaxyl and to apply it to the determination of its enantiomeric impurity (S-metalaxyl) in an enantiomerically pure agrochemical formulation in R-metalaxyl (metalaxyl-M), allowing for detection up to 1.2% for S-metalaxyl.

### 2.4. Nematicides

A nematicide is a type of chemical pesticide which is effective to eliminate nematodes parasitizing plants. One of the strategies to control phytonematode pests is the use of chemical nematicide compounds [81]. According to the literature reviewed, there is one article reporting a CE method for the simultaneous enantiomeric separation of an organophosphorus chiral nematicide, fenamiphos (with a chiral center), and its degradation metabolites in soil samples (fenamiphos sulfone (which has two chiral centers), and fenamiphos sulfoxide (which has a chiral center)) [49]. A dual CD system (CM-β-CD + HP-α-CD) at a pH of 5.0 under negative polarity (−20 kV) was employed. With the aim of increasing the solubility of these organophosphorus compounds, the influence of the addition of an organic modifier (MeOH, ethanol (EtOH), isopropanol (iPrOH), and ACN) to the BGE was investigated. The addition of 5% MeOH to the separation medium significantly improved the resolution values between fenamiphos sulfoxide diastereoisomers especially. The composition of the sample solvent was shown to be an important variable. Once the method was validated, the pesticides extracted from spiked soil samples were studied using two different methodologies: PLE with EtOH, EtOAc, or heptane as extraction solvents and SLE with MeOH as the extraction solvent. The recovery values obtained with both extraction techniques were similar (50–76% using PLE and 60–80% using SLE) but the extraction solvent volumes were twice as high when SLE was used. The method demonstrated its suitability to determine the fenamiphos enantiomeric degradation in soil samples.

### 2.5. Mixtures of Pesticides with Different Activity

Different studies were conducted on water, soils, or sludge samples, and in commercial agrochemical formulations prepared with mixtures of different pesticides with different activities. In all cases, the individual enantiomeric separation of each contaminant was achieved.

**Figure 4:**
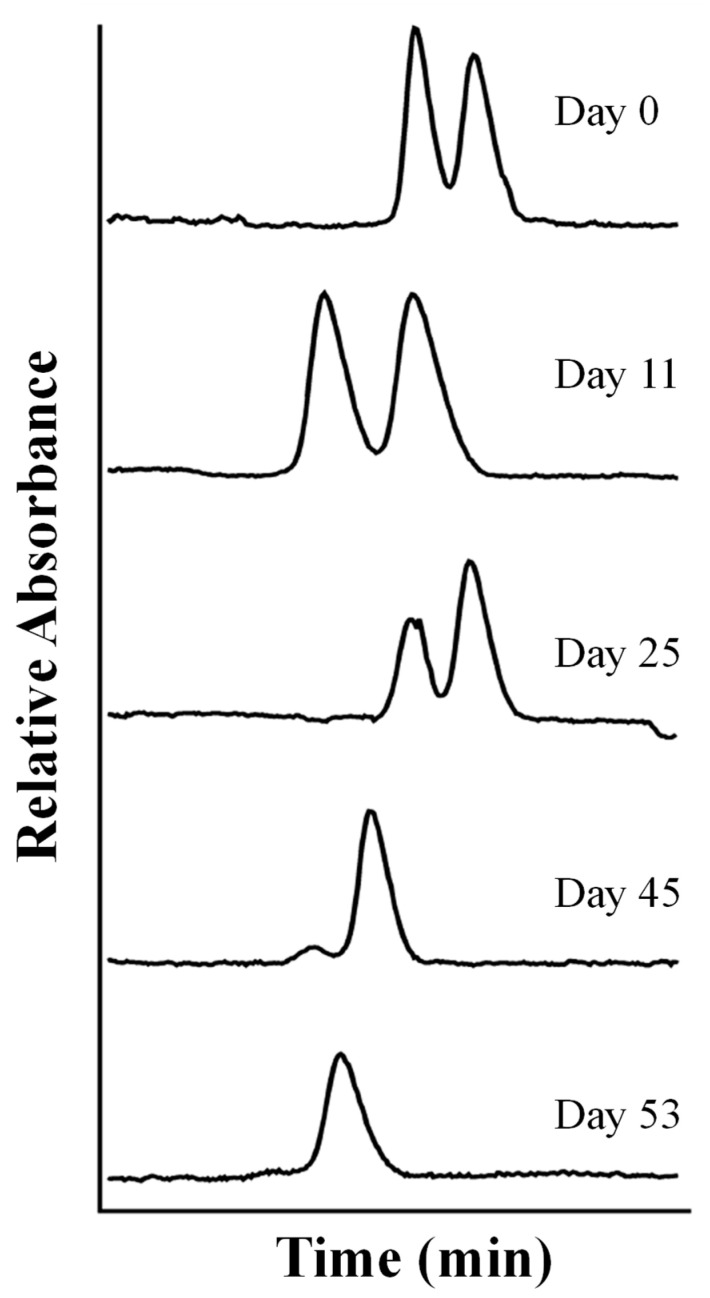
Electropherograms of metalaxyl enantiomers in a series of slurry samples revealing the change in the enantioselective degradation pattern with the incubation time. The first peak is R-(+)-metalaxyl. Migration times vary with time due to changes in the CE column EOF. Experimental conditions: BGE: 30 mM tetraborate buffer (pH 8.5) + 40 mM γ-CD + 100 mM SDS + ACN (15%, *v*/*v*), uncoated fused-silica capillary with an effective length of 75 µm id × 50 cm, hydrodynamic injection of × 5 s, temperature of 23 °C, applied voltage of +15 kV, and UV detection at 230 nm. Reproduced with permission [51].

Enantiomers of ruelene, also called crufomate (OP insecticide), and dichlorprop (a phenoxy acid herbicide) were analyzed in different soil samples by CE [50]. Each individual chiral separation was performed under different experimental conditions but at the same pH (pH 8.5). Since ruelene is a neutral compound, MEKC was the CE mode chosen, and a mixture of HP-β-CD, SDS, and ACN was employed as the BGE. However, the use of SDS was not required in the case of dichlorprop, which is an ionic compound, and TM-β-CD was used as the chiral selector in the presence of MeOH. Both enantiomers of ruelene exhibit insecticidal activity, although the (+)-enantiomer is four times more toxic. However, only (+)-dichlorprop exhibits herbicidal activity. The optimized CE methods were applied to investigate the influence of environmental changes on the degradation of the enantiomers of both compounds in different spiked soil samples collected from Brazil and Norway, which did not contain a high percentage of organic nutrients. For both compounds, deforestation in the Brazilian soils was shown to cause the transformation of the (−)-enantiomer, while the soil warming in Norway preferentially eliminated the active (+)-enantiomer. This study demonstrated that environmental changes might alter the persistence of the enantiomers of chiral contaminants. The separation of ruelene and dichlorprop was achieved in another work [62] using similar BGEs and detection modes as those in the previous work [50]. Under the respective optimized experimental conditions for each compound, the migration time for dichlorprop was 7.8 min, and for ruelene, it was around 13.5 min when analyzed at 25 µg mL^−1^. In addition to these two compounds, the individual baseline separation of bromochloroacetic acid (haloacetic water disinfectant) was also achieved using the same chiral selector as that for dichlorprop (TM-β-CD) at a pH of 8.5. The individual enantiomeric analysis of each pesticide in spiked samples was carried out (ruelene and dichlorprop in sludge samples and bromochloroacetic acid in river water samples) as well as a determination of enantiomeric fractions (EFs) (EF = area of the (+)-enantiomer divided by area of both enantiomers) as a function of the time to evaluate the microbial transformation. In the case of both ruelene and bromochloroacetic acid, the EF values decreased with time, demonstrating a loss of the (+)-enantiomer in both cases. However, in the case of dichlorprop, the opposite was true (the EFs increased since the concentration of the (−)-enantiomer decreased). In addition, the individual enantioseparation of five pesticides (including ruelene and dichlorprop, plus fonofos (also called diphonate), imazaquin, and metalaxyl) was reported by Jarman et al. using a different chiral CE method for each analyte [51], with the ruelene and dichlorprop separation methodologies based on a previous work [50]. Four different neutral CDs (γ-CD, HP-β-CD, heptakis (2,6-di-O-methyl)-β-cyclodextrin (DM-β-CD), TM-β-CD) were used as chiral selectors, although the addition of SDS as the micellar system was necessary in the case of neutral compounds (fonofos, ruelene, and metalaxyl). ACN was also added to the BGEs at percentages ranging from 15 to 20% (*v*/*v*). Tetraborate at a pH of 8.5 was the separation buffer for all the compounds except for imazaquin, for which acetate buffer at a pH of 4.5 was used. Enantiomeric resolution values varied from 1.2 to 2.0. The different chiral methodologies developed for the five fungicides were applied to degradation studies determining their EFs at various time intervals in different soils. The results showed that fonofos and imazaquin exhibited non-selective enantiomeric losses possibly due to non-selective or abiotic microbial reactions; ruelene and dichlorprop were selectively transformed showing the influence of environmental changes (as in the work of Lewis et al. [50]); and R-metalaxyl degraded more rapidly than S-metalaxyl, with an enantioselective transformation. Figure 4 shows, as an example, the electropherograms corresponding to metalaxyl enantiomers in different spiked slurry samples demonstrating that the enantioselective degradation pattern changed with the incubation time. To perform these studies, the spiked samples were subjected to different dissolution and dark-storage pretreatments.

The use of commercial surfactants alone or in combination with CDs was evaluated for the separation of enantiomers and isomers of seven pesticides (phenothrin, dimethomorph, bioallethrin, propiconazole, bitertanol, triadimenol, and fenpropathrin) [57]. Bitertanol has two chiral centers in its structure as propiconazole and triadimenol, as described above; bioalletrhin and fenpropathrin have only one chiral center; and phenothrin and dimethomorph have two geometric isomers (cis and trans isomers, and E and Z isomers, respectively). Two surfactants (SDS and SC, achiral and chiral, respectively) were assayed, with SDS being selected for the separation of fenpropathrin, propiconazole (the separation of both diastereoisomers), bitertanol (the separation of both diastereoisomers), and triadimenol the (separation of both diastereoisomers), while SC was used for the separation of bioallethrin, phenothrin (the separation of cis and trans isomers), and dimethomorph (the separation of E and Z isomers). Different CDs were employed: HP-β-CD for bioallethrin, γ-CD for fenpropathrin, DM-β-CD for phenothrin, TM-β-CD for propiconazole and bitertanol, and HP-γ-CD for triadimenol and dimethomorph. All the pesticides studied were resolved with resolution values ≥ 1.5 (with 7.1 for fenpropathrin and 8.7 for dimethomorph) with an analysis time from 6 to 18 min. In the case of bitertanol, the separation of its four enantiomers was achieved although they did not have good resolution values (1.1 between E_1_ and E_2_, and 1.2 between E_3_ and E_4_) when the chiral surfactant SC was added to the separation medium instead of SDS. The chiral methodologies developed were applied for the enantiomeric determination of the pesticides in lake water, with the spiked samples subjected to SPE with methyl tert-butyl ether (MTBE)/MeOH (90:10, *v*/*v*) as the eluent. The recovery values were in the range from 45 to 89% and the LODs were from 0.18 to 2.1 μg L^−1^, which are lower than or close to those required for many regulatory applications, including product registration.

The individual enantiomeric separation of 16 pesticides (insecticides, herbicides, and fungicides) was investigated by nano-LC and CEC [35]. Two chiral stationary phases, based on novel polysaccharides (cellulose tris(3-chloro-4-methylphenylcarbamate) (Sepapak-2) and cellulose tris(4-chloro-3-methylphenylcarbamate) (Sepapak-4)), were evaluated with both analytical techniques reaching the chiral separation of seven pesticides with Sepapak-2 and nine pesticides with Sepapak-4 under different experimental conditions. The stationary phase of Sepapak-4 was selected to compare both techniques using the same mobile phase (90:9:1, (*v*/*v*/*v*) ACN/H_2_O/ammonium formate at a pH of 2.5). The results showed that, for all the pesticides analyzed, the efficiency and the enantiomeric resolution were higher in CEC, although only eight pesticides were enantiomerically separated. The enantiomeric determination of metalaxyl by CEC was achieved in less than 15 min, and the relative limit of detection (RLOD) was below 0.6%. However, when a commercial formulation was analyzed, the enantiomeric impurity S-metalaxyl was determined to be above 3.7%. An analysis of samples (soil and tap water) spiked with a commercial formulation of metalaxyl-M was also carried out.

### 2.6. Antihypertensive Drugs

Antihypertensive drugs are employed to treat hypertension (high blood pressure) [82] in order to prevent some complications, such as stroke and myocardial infarction. β-adrenergic blocking agents, calcium channel antagonists, and angiotensin-converting enzyme (ACE) inhibitors are included in this group of drugs [83]. They are widely used, so they are found in relative high proportions in the aquatic environment. For example, metoprolol, propranolol, and atenolol are three of the chiral antihypertensive drugs that have been found at higher concentrations in fresh surface water samples (4, ≈3, and 4 µg L^−1^, respectively), as well as in marine and estuarine surface waters (≈2.5, 2, and ≈2.7 µg L^−1^, respectively) [84].

Silva et al. reported the enantiomeric separation of antihypertensive drugs in different aquatic media. Thus, the simultaneous separation of four pairs of enantiomers of the β-blockers atenolol, propranolol, metoprolol, and pindolol was carried out by CE with a BGE composed of methyl-β-cyclodextrin (M-β-CD) as the chiral selector at an acidic pH in order to maintain the charge of the drugs (with pK_a_ values from 9.45 to 9.70) [58]. The effect of different experimental variables, such as the M-β-CD concentration, temperature, separation voltage, injection time, and buffer concentration, on the separation was studied. Under optimized conditions, the separation of the four pairs of enantiomers was possible in 35 min, with enantiomeric resolutions of 1.45 for pindolol, 1.12 for atenolol, 2.85 for propranolol, and 1.25 for metoprolol. The developed method was applied to the analysis of the drugs in spiked river, tap, and groundwater samples. Sample treatment by off-line SPE with two different types of sorbents (mesoporous silica functionalized with octadecyl groups (SBA_15_-C_18_) (obtained by a post-synthesis method) and commercial amorphous silica C_18_ (ExtraBondR C_18_)) was compared, employing only tap water for the optimization of the SPE procedure and the validation of SPE-CE method. A preconcentration factor of 300 was reached using 100 mg of SBA_15_-C_18_ as the sorbent, with good levels of repeatability and recovery ranging from 96 to 105%. Worse extraction capacities and lower recoveries were obtained with a commercial C_18_ sorbent. The analytical characteristics of the SPE-CE method were adequate within quantification limits ranging from 5.3 to 13.7 µg L^−1^. The SBA_15_-C_18_ sorbent was selected for the extraction of the enantiomers of β-blockers from spiked tap, river, and groundwater samples with recoveries from 58.0 to 105%. The SPE-CE method was improved upon by the same authors in another article [59]. In that work, they modified the electrophoretic conditions by changing the capillary length, the temperature, and the injection conditions (see Table 1). The influence of the alkyl chain length of the sorbent was evaluated by comparing C_3_, C_8_, and C_18_. After evaluating the three types of sorbents, the results showed that both 100 mg of C_8_ and C_18_ resulted in the best recovery values in 100 mL of spiked tap water samples. Then, the effect of the amount of sorbent (100 and 200 mg) and the sample loading volume used (100–250 mL) was evaluated. The best results were obtained with 200 mg of the C_8_ sorbent and 250 mL of the spiked sample with recovery values of 80% for all the enantiomers and a preconcentration factor of 500, except for atenolol, for which a preconcentration factor of 300 was obtained using 100 mg of the C_18_ sorbent with 100 and 150 mL of the spiked sample. Although an increase in the analysis time of 7 min was obtained (the total analysis time was 42 min), the sensitivity of the method was improved by decreasing the LOD values by more than half for each of the analytes with respect to those of the previous methodology.

### 2.7. Anti-Inflammatory Drugs

Numerous anti-inflammatory drugs, such as ibuprofen, naproxen, ketoprofen, and flurbiprofen, and some of their metabolites are chiral. Only ketoprofen and ibuprofen have been enantiomerically determined in water samples by CE. For ketoprofen, a novel on-line preconcentration method by the electrokinetic accumulation of ketoprofen enantiomers at the pH boundary followed by enantioselective mobilization by MEKC was reported [61]. The enantioselective mobilization was performed with a mixture of SDS, S-β-CD, and TM-β-CD. Under optimized conditions, the determination of ketoprofen enantiomers at nanomolar levels was successfully achieved in spiked wastewater samples after using a simple method of filtration as a clean-up step with recovery values from 91 to 94%. The LODs obtained were 0.64 µg L^−1^ and 0.86 µg L^−1^, which indicate enhancement factors of 9921 and 8529, respectively. For ibuprofen, a dispersive SPE (d-SPE) followed by a CE-UV analysis was proposed for the preconcentration, sample cleaning, and chiral separation of ibuprofen at trace levels in environmental and biological matrices (water and human urine) [39]. An experimental design was implemented to evaluate the effect of the main variables influencing the extraction recovery and enantiomeric resolution. The best enantioseparation of ibuprofen was observed with 1 mM VC as the chiral selector in a phosphate buffer (at a pH of 6.5) in around 26 min. Under optimal conditions of the d-SPE using molybdenum disulphide (MoS_2_) as the novel sorbent, a preconcentration factor of 33.4 was obtained. The validated method was applied to the determination of ibuprofen in spiked samples with recovery values of 93% for water and 94.3% for human urine. The method developed in this work showed the lowest LODs and similar recovery values with respect to those of other methods reported in the literature based on the use of solid-phase microextraction (SPME)-LC [85], SPE-CE [86,87], and LLE-GC-MS [88]. In addition, this method can be employed for the analysis of water samples since the LODs are below the concentration at which the drug was found in this work [17].

### 2.8. Mixtures of Different Drug Families

**Figure 5:**
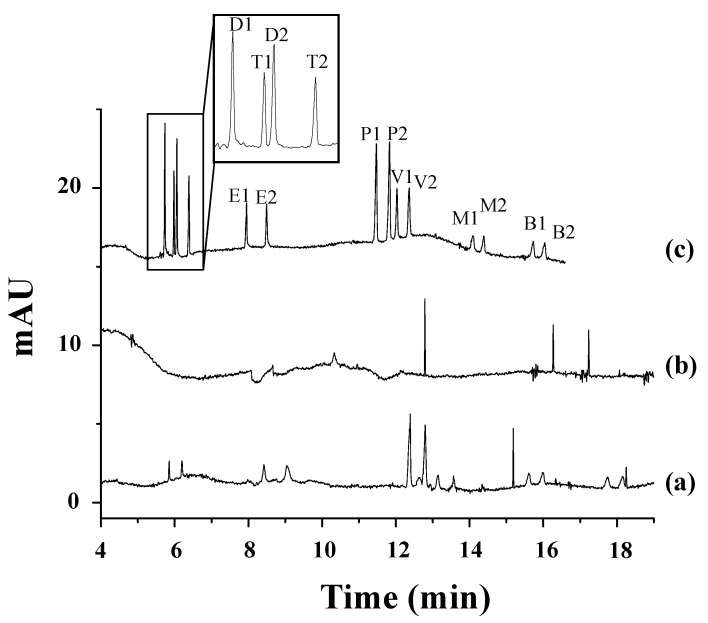
Electropherograms corresponding to the enantiomeric separation of (a) wastewater sample 1 spiked before SPE treatment with the seven racemic drugs at the following enantiomeric concentrations: D and P (2.5 µg L^−1^), E (8.2 µg L^−1^), T and V (5.0 µg L^−1^), M and B (6.2 µg L^−1^), using 100 mg of PMO-TESB-1 sorbent and 200 mL of sample volume; (b) non-spiked wastewater sample 1 after SPE treatment; and (c) a standard solution containing the seven racemic drugs at concentrations of 20 mg L^−1^ for terbutaline and econazole and 10 mg L^−1^ for the other. D: duloxetine, T: terbutaline, E: econazole, P: propranolol, V: verapamil M: metoprolol, B: betaxolol; 1, First-migration enantiomer; 2, Second-migration enantiomer. Experimental conditions: BGE: 25 mM phosphate buffer (pH of 3.0) + 2% (*w*/*v*) S-β-CD, uncoated fused-silica capillary with effective length of 50 µm i.d. *×* 50 cm, injection by pressure of 50 mbar *×* 10 s, temperature of 20 °C, applied voltage of −20 kV, and UV detection at 210 ± 5 nm. Reproduced with permission [53].

The simultaneous enantiomeric separation by CE of chiral drugs belonging to different families and their determination in water samples after preconcentration by SPE was reported [53]. This work described a simple method of synthesis, in only one step, for the preparation of new periodic mesoporous organic materials with a neutral phenylene-bridged ligand as SPE sorbents for the extraction of seven drugs with different pharmacological activities (duloxetine, terbutaline, econazole, propranolol, verapamil, metoprolol tartrate, and betaxolol) from wastewater samples. The effects of combining two silica sources to prepare mesoporous sorbents and of the existence of an alkyl chain joined to the benzene ring on the performance of the materials were investigated as well. Under the optimized SPE conditions, recoveries between 80.5 and 103.1% (except for that of terbutaline) and preconcentration factors of 400 with a good level of repeatability were obtained. The simultaneous enantiomeric separation of the seven drugs was possible using S-β-CD in 16 min with enantiomeric resolutions ranging from 2.4 to 8.5. An analysis of spiked wastewater samples from different treatment plants (see Figure 5) showed recoveries between 73.9 and 102.9%, except for econazole (which had recovery values between 58.5 and 72.4%). The authors demonstrated the potential of periodic mesoporous organosilica materials as sorbents for off-line SPE prior to CE separation for simultaneous chiral analyses of drugs in wastewater samples.

## 3. Applications of Chiral CE to Toxicity Studies

Table 2 groups the articles reporting stability and toxicity studies with aquatic non-target organisms for different chiral pollutants (pesticides, drugs, and cosmetics) using CE. The classification of contaminants according to their toxicity for aquatic ecosystems is governed by European Directive 93/67/EEC [89]. The CE separation modes employed in these cases were CD-EKC and CD-MEKC. The latter mode was selected as the separation mode in one of these works due to the low solubility of the compound using a mixture of the bile salt SDC and a neutral CD (HP-β-CD) [20]. CD-EKC with anionic CDs (Succ-β-CD, S-β-CD, S-γ-CD, and carboxyethyl-β-cyclodextrin (CE-β-CD)) was chosen in the other works, which are reported as shown in Table 2. In addition, the use of a chiral ionic liquid as an additive in the separation medium in combination with a CD was studied in the most recent work describing the enantioseparation of ibrutinib [90]. The different non-target organisms for which stability and ecotoxicity studies have been carried out are aquatic plants (*Spirodela polyrhiza*), marine bacteria (*Vibrio fischeri*), and microcrustaceans (*Daphnia magna*) (see Table 2). The incubation of these different non-target organisms as turions in the case of plants [2,18,19] or eggs in the case of microcrustaceans [16,20,90], under continuous light irradiation with a controlled light intensity at 6000 lux and static conditions at temperatures ranging from 20 to 25 °C, was required. However, the freeze-dried marine bacterium was rehydrated with a reconstitution solution in order to prepare the bacterial inoculum [19,21]. Stability and toxicity studies in the presence of the emerging contaminants required the grown plants to be incubated with continuous illumination at 6000 lux from 72 to 96 h and microcrustaceans to be incubated under darkness from 48 to 96 h, except for the bacterium, for which its bioluminescence inhibition was measured after 15 min (for the first time) and up to 1 h.

As shown in Table 2, stability and ecotoxicity studies have only been published for two chiral pesticides, sulfoxaflor [19] and tetramethrin [20].

**Figure 6:**
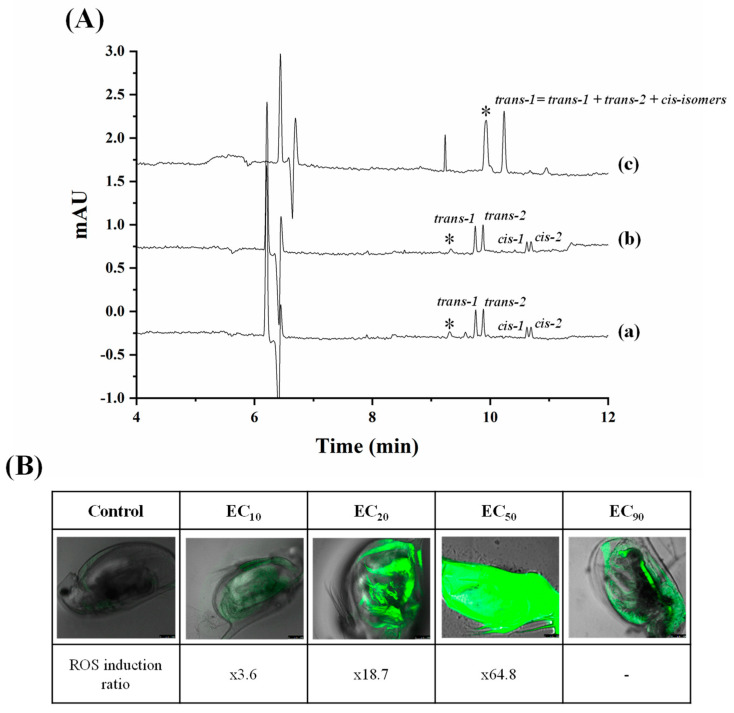
(**A**) Analysis of tetramethrin (a) standard solution, under (b) abiotic and (c) biotic (in presence of daphnids) conditions, respectively, at 72 h. Experimental conditions: BGE: 100 mM borate buffer (pH 8.0) + 15 mM HP-β-CD + 50 mM SDC, uncoated fused-silica capillary with effective length of 50 µm i.d. × 50 cm, injection by pressure of 50 mbar × 2 s, temperature of 15 °C, applied voltage of +20 kV, and UV detection at 220 nm. * = unknown peak corresponding to the medium. (**B**) Confocal images of ROS generation in *Daphnia magna* at 24 h of incubation at different effect levels of tetramethrin (EC_10_, EC_20_, EC_50_, EC_90_) and control. Reproduced with permission [20].

The studies on the stability and toxicity of sulfoxaflor under abiotic and biotic conditions were carried out in two non-target aquatic organisms, a freshwater plant (*Spirodela polyrhiza*) and the marine bacterium *Vibrio fischeri*, using the chiral CE methodology described in the previous section [19]. Only the effect of the mixture of the four stereoisomers of sulfoxaflor could be studied because individual stereoisomers were not commercially available. The stability studies revealed that after 96 h, under biotic conditions and in the presence of the freshwater plant, a decay percentage of 15% was observed, while under abiotic conditions, the decay was 3%. However, after 1 h under biotic conditions in the presence of the marine bacterium, a decay rate of 31% was observed, while under abiotic conditions, it was 11%. Then, the stability of sulfoxaflor is low in marine water compared to freshwater. In terms of toxicity, according to the data obtained for the effective concentration beginning at a level of 50% inhibition (EC_50_), it was classified as a very toxic compound for the continental aquatic environment. The developed CD-MEKC methodology that enabled the chiral separation of the stereoisomers of tetramethrin was applied to the study of their stability at the enantiomeric level and to the determination of the ecotoxicological parameters against the microcrustacean *Daphnia magna* [20]. Studies of each individual stereoisomer could not be achieved because they were not commercially available. Figure 6A shows, as an example, the analysis of tetramethrin under abiotic and biotic conditions at 72 h under the optimized experimental CE conditions. Under abiotic conditions, the compound was stable. However, 95% was transformed under biotic conditions by an isomerization process from the *trans*-2-isomer and *cis*-isomer to the *trans*-1-isomer. The remaining 5% could be bioaccumulated in the body of the microinvertebrate *Daphnia magna*. In addition, the results showed the first evidence of an oxidative-stress-mediated (ROS) mode of action for tetramethrin on aquatic microinvertebrates, which is focalized to the digestive track. As the EC_50_ value (1.8 mg L^−1^) indicates that tetramethrin is toxic to non-target aquatic organisms, this toxicity could be attributed to the *trans*-1-isomer. This effect can be observed in Figure 6B which shows the confocal images of ROS generation in *Daphnia magna* at 24 h of incubation at different tetramethrin effect levels and control.

As already explained in the introduction of this article, the widespread use of pharmaceuticals has caused them to be considered as emerging pollutants due to their appearance in environmental samples. Many works have reported the presence of these compounds in the environment, especially in aquatic ecosystems. However, although biota is also affected, not much attention has been paid to evaluate the impact that these emerging contaminants can cause in the metabolism of non-target organisms [91]. A CE method was developed for the simultaneous enantiomeric separation of two chiral drugs, duloxetine (antidepressant) and econazole (antifungal) [2,16]. This work is of interest since emerging contaminants are not isolated in the environment and are able to present synergistic or antagonistic effects, as mentioned above. The developed method was applied for the first time to the study of the stability of each drug and a mixture of both, as well as their toxicity towards two different non-target organisms, the aquatic plant *Spirodela polyhiza* [2] and the microcrustacean *Daphnia magna* [16]. In both cases, standard racemate dilutions were evaluated for both chiral compounds. The method was based on the use of phosphate buffer (at a pH of 3.0) and S-β-CD (1.5%, *w*/*v*) as a chiral selector. Simultaneous chiral separation was achieved in 7.5 min, reaching resolution values of 7.9 for duloxetine enantiomers and 6.5 for econazole enantiomers. Stability evaluations for the racemates and the enantiomers on *Spirodela polyhiza* allowed researchers to observe differences in the decay percentages for both drugs and this is true at individual level as well as in binary mixtures. For individual solutions and under abiotic conditions (in the absence of aquatic plants), both duloxetine and econazole decomposed (with decay percentages of 80 and 60%, respectively) [2]. Under biotic conditions (in the presence of non-target organisms), econazole showed a similar stability to that observed under abiotic conditions. However, duloxetine increased its decay percentage. When both drugs were mixed, duloxetine increased its decay percentages under biotic conditions while econazole decomposed by 100% under both abiotic and biotic conditions. To determine, for the first time, the toxicity of these drugs on *Spirodela polyrhiza*, the EC_50_ values were determined for each of the compounds studied and for the mixture, and it was concluded that they are very toxic compounds, although econazole showed more toxicity than duloxetine and the binary mixture. Stability studies carried out with *Daphnia magna* revealed that the duloxetine concentration did not appreciably vary under abiotic and biotic conditions in solutions containing this drug alone. However, after 72 h of incubation, stability profiles for racemic duloxetine and each of its enantiomers were different in mixtures of duloxetine and econazole [16]. Depending on the initial nominal concentrations, values from 46 to 77% were obtained as decay percentages for duloxetine enantiomers. Econazole disappeared at 100% both in the individual solutions and in the mixture. In addition, the toxicity of the mixture of both drugs was investigated for the first time in this work and it was found to be more toxic than single solutions of the drugs after 48 h of incubation. In the last year, Amariei et al. published another work in which the stability and ecotoxicity for ivabradine individual enantiomers and their racemic mixture were evaluated in the *Vibrio fischeri* marine bacterium [21]. For this purpose, the chiral method developed by Casado et al. [92] and was employed, as described in Table 2. This drug did not show any enantiomeric interconversion, presenting a moderate chiral stability under biotic conditions. However, the inhibition of bacterial bioluminescence was enantioselectively affected; i.e., depending on whether R-, S-, or racemic ivabradine was present in the biotic samples, different results were obtained. The EC_50_ ecotoxicity values were 75.98, 11.11, and 7.93 mg L^−1^ for R-ivabradine, racemic ivabradine, and S-ivabradine, respectively. Moreover, oxidative stress observed under confocal microscopy showed that S-ivabradine was the main mechanism of drug toxicity, so, according to the authors, it is very important to perform further ecotoxicity studies in different aquatic microorganisms to know the risk posed by the administration of the drug. Recently, García-Cansino et al. evaluated the stability and ecotoxicity of both R,S-ibrutinib and R-ibrutinib in the aquatic microorganism *Daphnia magna* [90]. For this purpose, they developed two chiral methodologies using CD-EKC, one based on the use of S-γ-CD as a chiral selector and another methodology in which CD was combined with the chiral ionic liquid [TMA][L-Lys] as an additive to the separation medium. This additive was added to improve the enantioresolution value, which increased from 1.5 to 3.3, although the analysis time also increased by 3.9 min. In this case, R,S- and R-ibrutinib were found to be stable under abiotic conditions. However, R,S-ibrutinib and R-ibrutinib showed a 20% reduction in their concentrations under biotic conditions after 24 h of incubation in the dark, employing both separation methods, and were higher after 48 h (45% for R,S- and 30% for R-ibrutinib). After evaluation of the ecotoxicity of both (R,S-ibrutinib and R-ibrutinib) after 24 and 48 h, they could be categorized as toxic according to their EC_50_ values after 48 h (4 and 4.9 mg L^−1^, respectively, using the single separation system, and 4 and 4.8 mg L^−1^, respectively, using the dual separation system).

**Figure 7:**
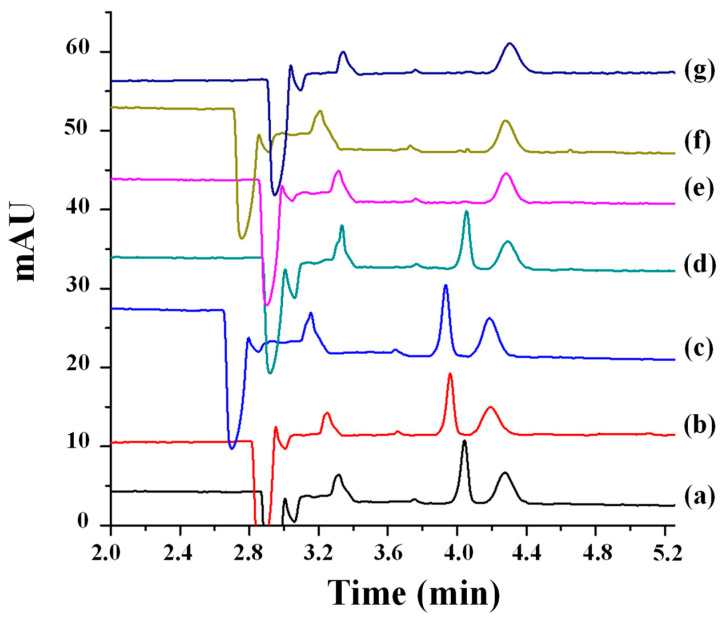
Electropherograms obtained at (a) 0 h of exposure to racemic panthenol (200 mg L^−1^) under abiotic conditions (culture medium); at 96 h exposure to racemic panthenol (200 mg L^−1^) under (b) biotic (plant samples) and abiotic conditions (c) with and (d) without light; and at 96 h exposure to dexpanthenol (100 mg L^−1^) under (e) biotic (plant samples) and abiotic conditions (f) with and (g) without light. Experimental conditions: BGE: 100 mM borate buffer (pH 9.0) + 25 mM CE-β-CD, uncoated fused-silica capillary with effective length of 50 µm i.d. × 50 cm, injection by pressure of 50 mbar × 10 s, temperature of 30 °C, applied voltage of +30 kV, and UV detection at 205 ± 30 nm. Reproduced with permission [18].

Finally, a cosmetic has been studied by CE as an emerging chiral pollutant. Panthenol (provitamin B_5_) is the alcohol of pantothenic acid (vitamin B_5_). It has two enantiomers, both with moisturizing properties. However, while D-panthenol (dexpanthenol) is the biologically active enantiomer, L-panthenol is inactive [93]. Thus, it is used in pharmaceutical, personal care, and cosmetic products [94,95]. Panthenol was enantiomerically separated by CD-EKC in 4.2 min with a resolution value of 2.0 using CE-β-CD as the chiral selector [18]. The chiral method was applied to study the stability of racemic panthenol and D-panthenol under abiotic and biotic conditions and their toxicity on the aquatic plant *Spirodela polyrhiza*. Figure 7 depicts the electropherograms corresponding to 0 h under abiotic conditions and after 96 h of incubation with racemic panthenol and dexpanthenol under biotic and abiotic conditions with and without light. The concentrations did not appreciably vary under any of the experimental conditions studied. The results showed that, regardless of the experimental conditions, both compounds were stable with only a decay rate of less than 5% for L-panthenol and 9% for D-panthenol (both in the racemic mixture and in the single enantiomer). For the first time, it was demonstrated that both compounds are toxic. However, the results revealed that the toxicity of racemic panthenol is considerably higher than that of dexpanthenol for short exposure times (24 h) and their toxicity differences are smaller at larger exposure times.

## 4. Conclusions

The characteristics and applications of the analytical methodologies developed by CE for the stereoselective determination of pesticides and emerging contaminants in environmental (water and soil) and food samples, as well as pesticides in agrochemical formulations are reviewed in this article. The most frequently used CE separation mode has been EKC, whereas CEC and NACE have been employed to a lesser extent (few articles have been published based on these CE separation modes). CDs have been by far the most widely used chiral selectors for the separation of chiral pesticides and emerging contaminants. Among the different CDs employed, TM-β-CD, γ-CD, HP-β-CD, and HP-γ-CD can be highlighted as the preferred ones. VC and bile salts have also been used as chiral selectors alone or in combination with CDs. Other additives such as urea or organic modifiers (MeOH, ACN) have also been employed to improve separations. UV detection was mainly employed in the works reviewed, although the use of fluorescence detection has also been described. For sample treatment, SPE has been the most frequently used with respect to LLE and PLE, although the dissolution of samples in organic solvents has also been widely used. Other solvents such as water or a mixture of urea and SDC have also been used. On-line preconcentration techniques, such as large volume sample stacking or sweeping, have been implemented to increase the necessary sensitivity to analyze environmental and food samples. The lowest limits of detection obtained corresponded to the use of off-line SPE with UV detection (0.18–0.98 µg L^−1^), LVSS as the on-line preconcentration technique and fluorescence detection (0.47 µg L^−1^), and on-line preconcentration by electrokinetic accumulation and UV detection (0.64 µg L^−1^). In numerous works, the individual enantiomeric separation of more than one compound (with similar or different chemical natures) has been carried out, and in some of these, the simultaneous enantiomeric separation of the investigated compounds was performed. The methodologies developed were mainly applied for the analysis of chiral pollutants in environmental (water and soil) samples and in food, as well as for the determination of pesticides in commercial agrochemical formulations. Applications for the study of the enantioselective degradation of some contaminants in environmental samples have also been reported. Stability and toxicity studies have been carried out for pesticides and for emerging contaminants (pharmaceuticals and cosmetics). Toxicity studies have involved non-target aquatic organisms, such as aquatic plants (*Sorghum bicolor* and *Spirodela polyrhiza*), microcrustaceans (*Daphnia magna*), and marine bacteria (*Vibrio fischeri*). In these cases, chiral CE enabled researchers to determine the real concentrations of contaminants at the enantiomeric level for the first time. All the reported works show the relevance of developing analytical methodologies to achieve the analysis of samples of environmental concern, to assess the quality of agrochemical formulations, and to contribute to the real evaluation of the stability and toxicity of agrochemicals and emerging pollutants.

## Figures and Tables

**Table 2:** Applications of CE for the evaluation of the stability and toxicity of chiral pollutants on non-target organisms.

Analyte (*Chemical family*)	Applications	Sample Treatment	Separation Conditions	Analysis Time	LOD (Rs)	Ref.
Sulfoxaflor *(Sulfoximine)*	Stability evaluation of the stereoisomers under biotic and abiotic conditions on aquatic plant *Spirodela polyrhiza* and the marine bacterium *Vibrio fischeri.*	Turions for *Spirodela polyrhiza* were germinated in a growth medium for 3 days (25 °C). Freeze-dried bacterium *Vibrio fischeri* was reactivated in NaCl solution under the indications of the BioTox™ kit for 15 min. Exposure experiments were conducted with the analyte for 96 h and 1 h, respectively.	BGE: 100 mM borate buffer, pH 9.0 + 15 mM Succ-β-CD Capillary: 50 µm i.d. × 50 cm e.l.; T^a^: 15 °C; V: +20 kV; Injection: 50 mbar × 8 s; Detection: UV 205 ± 30 nm	13.8 min	E_1_: 0.9 mg L^−1^ E_2_: 1.0 mg L^−1^ E_3_: 0.9 mg L^−1^ E_4_: 0.9 mg L^−1^ (E_1_, E_2_: 2.1 E_2_, E_3_: 1.5 E_3_, E_4_: 2.6)	[19]
Tetramethrin *(Pyrethroid)*	Stability and toxicity evaluation under biotic and abiotic conditions on microcrustacean *Daphnia magna*.	*Daphnia magna* eggs were incubated in a growth medium for 3 days (20–22 °C). Exposure experiments were conducted with the analyte for 72 h.	BGE: 100 mM borate buffer, pH 8.0 + 15 mM HP-β-CD + 50 mM SDC Capillary: 50 µm i.d. × 50 cm e.l.; T^a^: 15 °C; V: +20 kV; Injection: 50 mbar × 2 s; Detection: UV 220 ± 4 nm	<12.5 min	*Trans*-tetramethrin: 1.30 mg L^−1^ (1.7) *Cis*-tetramethrin: 0.97 mg L^−1^ (1.3)	[20]
1- Duloxetine *(Amine)* 2- Econazole *(Imidazole)*	Stability and toxicity studies under biotic and abiotic conditions on aquatic plant *Spirodela polyrhiza.*	Turions were germinated in a growth medium for 3 days (25 °C). Exposure experiments were conducted with the analytes for 72 h.	BGE: 25 mM phosphate buffer, pH 3.0 + S-β-CD (1.5%, *w*/*v*) Capillary: 50 µm i.d. × 50 cm e.l.; T^a^: 30 °C; V: −20 kV; Injection: 50 mbar × 10 s; Detection *: UV; 1- 220 ± 5 nm; 2- 200 ± 5 nm	7.5 min	1- E_1_: 0.2 mg L^−1^ E_2_: 0.3 mg L^−1^ (7.9) 2- E_1_: 0.7 mg L^−1^ E_2_: 0.8 mg L^−1^ (6.5)	[2]
1- Duloxetine *(Amine)* 2- Econazole *(Imidazole)*	Stability and toxicity evaluation under biotic and abiotic conditions on microcrustacean *Daphnia magna*.	*Daphnia magna* eggs were incubated for 3 days (20 ± 1 °C). Exposure experiments were conducted with the analytes for 48 h.	BGE: 25 mM phosphate buffer, pH 3.0 + S-β-CD (1.5%, *w*/*v*) Capillary: 50 µm i.d. × 50 cm e.l.; T^a^: 30 °C; V: −20 kV; Injection: 50 mbar × 10 s; Detection *: UV; 1- 220 ± 5 nm; 2- 200 ± 5 nm	7.5 min	1- E_1_: 0.3 mg L^−1^ E_2_: 0.4 mg L^−1^ (7.9) 2- E_1_: 1.0 mg L^−1^ E_2_: 1.1 mg L^−1^ (6.5)	[16]
Ivabradine *(Benzazepine)*	Stability and toxicity evaluation under biotic and abiotic conditions on marine bacterium *Vibrio fischeri*.	Freeze-dried bacterium *Vibrio fischeri* was reactivated in NaCl solution under the indications of the BioTox™ kit for 15 min. Exposure experiments were conducted with the analyte for 1 h.	BGE: 50 mM formate buffer, pH 2.0 + 4 mM S-γ-CD Capillary: 50 µm i.d. × 50 cm e.l.; T^a^: 25 °C; V: −30 kV; Injection: 50 mbar × 5 s; Detection: 200 nm	6 min	R-ivabradine: 0.11 mg L^−1^ S-ivabradine: 0.11 mg L^−1^ (2.7)	[21]
Ibrutinib	Stability and toxicity evaluation under biotic and abiotic conditions on microcrustacean *Daphnia magna*.	*Daphnia magna* eggs were incubated for 3 days (20 ± 2 °C). Exposure experiments were conducted with the analytes for 48 h.	Method 1: BGE: 25 mM formate buffer, pH 3.0 + 2 mM S-γ-CD Method 2: BGE: 25 mM formate buffer, pH 3.0 + 2 mM S-γ-CD + 5 mM [TMA][L-Lys] Capillary: 50 µm i.d. × 50 cm e.l.; T^a^: 30 °C; V: −30 kV; Injection: 50 mbar × 5 s; Detection: 200 ± 4 nm	Method 1: 4.2 min Method 2: 8.1 min	Method 1: 0.1 mg L^−1^ (1.5) Method 2: 0.1 mg L^−1^ (3.3)	[90]
Panthenol *(Provitamin)*	Stability and toxicity evaluation under biotic and abiotic conditions on aquatic plant *Spirodela polyrhiza*.	Turions were germinated in a growth medium for 3 days (23 ± 2 °C). Exposure experiments were conducted with the analyte for 96 h.	BGE: 100 mM borate buffer, pH 9.0 + 25 mM CE-β-CD Capillary: 50 µm i.d. × 50 cm e.l.; T^a^: 30 °C; V: +30 kV; Injection: 50 mbar × 10 s; Detection: UV 205 ± 30 nm	4.2 min	L-panthenol: 1 mg L^−1^ D-panthenol: 4 mg L^−1^ (2.0)	[18]

BGE: background electrolyte; CE-β-CD: carboxyethyl-β-cyclodextrin; E_1_: first-migration enantiomer; E_2_: second-migration enantiomer; E_3_: third-migration enantiomer; E_4_: fourth-migration enantiomer; e.l.: effective length; HP-β-CD: 2-hydroxypropyl-β-cyclodextrin; i.d.: internal diameter; LOD: limit of detection; MeOH: methanol; NaCl: sodium chloride; Rs: resolution; S-β-CD: sulphated-β-cyclodextrin; S-γ-CD: sulphated-γ-cyclodextrin; SDC: sodium deoxycholate; Succ-β-CD: succinyl-β-cyclodextrin; UV: ultraviolet. * 210 nm was also employed as intermediate wavelength for binary mixtures of both compounds.

## References

[1] Ye J., Zhao M., Niu L., Liu W. (2015). Enantioselective environmental toxicology of chiral pesticides. Chem. Res. Toxicol..

[2] Valimaña-Traverso J., Amariei G., Boltes K., García M.A., Marina M.L. (2019). Stability and toxicity studies for duloxetine and econazole on Spirodela polyrhiza using chiral capillary electrophoresis. J. Hazard. Mater..

[3] Casado N., Valimaña-Traverso J., García M.A., Marina M.L. (2020). Enantiomeric determination of drugs in pharmaceutical formulations and biological samples by Electrokinetic Chromatography. Crit. Rev. Anal. Chem..

[4] McConathy J., Owens M.J. (2003). Stereochemistry in Drug Action. Prim. Care Companion J. Clin. Psychiatry.

[5] Aboul-Enein H.Y., Ali I. (2003). Chiral Separations by Liquid Chromatography and Related Technologies.

[6] Röesner M., Capraro H.G., Jacobson A.E., Atwell L., Brossi A., Lorio M.A., Williams T.H., Chignell C.F. (1981). Biological effects of modified colchicines. Improved preparation of 2-demethylcolchicine, 3-demethylcolchicine, and (+)-colchicine and reassignment of the position of the double bond in dehydro-7-deacetamidocolchicines. J. Med. Chem..

[7] Del Bubba M., Checchini L., Lepri L. (2013). Thin-layer chromatography enantioseparations on chiral stationary phases: A review. Anal. Bioanal. Chem..

[8] Ariëns E. (1991). Racemic therapeutics-ethical and regulatory aspects. Eur. J. Clin. Pharmacol..

[9] Purushoth P. (2016). Pharmaceutical review and its importance of chiral chromatography. Int. J. Res. Pharm. Chem..

[10] Bertaso A., Musile G., Gottardo R., Seri C., Tagliaro F. (2015). Chiral analysis of methorphan in opiate-overdose related deaths by using capillary electrophoresis. J. Chromatogr. B Anal. Technol. Biomed. Life Sci..

[11] García-Martín E., Martínez C., Tabarés B., Frías J., Agúndez J.A.G. (2004). Interindividual variability in ibuprofen pharmacokinetics is related to interaction of cytochrome P4502C8 and 2C9 amino acid polymorphisms. Clin. Pharmacol. Ther..

[12] Hammami R., Nouira I., Frein Y. (2018). Effects of customers’ environmental awareness and environmental regulations on the emission intensity and price of a product. Decis. Sci..

[13] Rentsch K.M. (2002). The importance of stereoselective determination of drugs in the clinical laboratory. J. Biochem. Biophys. Methods.

[14] Garrison A.W. (2006). Probing the enantioselectivity of chiral pesticides. Environ. Sci. Technol..

[15] Lucci E., Dal Bosco C., Antonelli L., Fanali C., Fanali S., Gentili A., Chankvetadze B. (2022). Enantioselective high-performance liquid chromatographic separations to study occurrence and fate of chiral pesticides in soil, water, and agricultural products. J. Chromatogr. A.

[16] Valimaña-Traverso J., Amariei G., Bolter K., García M.Á., Marina M.L. (2019). Enantiomer stability and combined toxicity of duloxetine and econazole on Daphnia magna using real concentrations determined by capillary electrophoresis. Sci. Total Environ..

[17] Camacho-Muñoz D., Martín J., Santos J.L., Aparicio I., Alonso E. (2014). Concentration evolution of pharmaceutically active compounds in raw urban and industrial wastewater. Chemosphere.

[18] Jiménez-Jiménez S., Amariei G., Boltes K., García M.A., Marina M.L. (2021). Enantiomeric separation of panthenol by Capillary Electrophoresis. Analysis of commercial formulations and toxicity evaluation on non-target organisms. J. Chromatogr. A.

[19] Jiménez-Jiménez S., Amariei G., Boltes K., García M.A., Marina M.L. (2021). Stereoselective separation of sulfoxaflor by electrokinetic chromatography and applications to stability and ecotoxicological studies. J. Chromatogr. A.

[20] Greño M., Amariei G., Boltes K., Castro-Puyana M., García M.A., Marina M.L. (2021). Ecotoxicity evaluation of tetramethrin and analysis in agrochemical formulations using chiral electrokinetic chromatography. Sci. Total Environ..

[21] Amariei G., Jiménez-Jiménez S., García M.A., Marina M.L., Boltes K. (2022). First eco-toxicological evidence of ivabradine effect on the marine bacterium Vibrio fischeri: A chiral view. Sci. Total Environ..

[22] Tůma P. (2023). Progress in on-line, at-line, and in-line coupling of sample treatment with capillary and microchip electrophoresis over the past 10 years: A review. Anal. Chim. Acta..

[23] de Rijke E., Out P., Niessen W.M.A., Ariese F., Gooijer C., Brinkman U.A.T. (2006). Analytical separation and detection methods for flavonoids. J. Chromatogr. A.

[24] Kodama S., Saito Y., Chinaka S., Yamamoto A., Hayakawa K. (2006). Chiral capillary electrophoresis of agrochemicals in real samples. J. Health Sci..

[25] Herrero M., Simó C., García-Cañas V., Fanali S., Cifuentes A. (2010). Chiral capillary electrophoresis in food analysis. Electrophoresis.

[26] Hernández-Borges J., Rodríguez-Delgado M.A., García-Montelongo F.J. (2005). Chiral analysis of pollutants and their metabolites by capillary electromigration methods. Electrophoresis.

[27] Pérez-Fernández V., García M.A., Marina M.L. (2011). Chiral separation of agricultural fungicides. J. Chromatogr. A.

[28] Eash D.T., Bushway R.J. (2000). Herbicide and plant growth regulator analysis by capillary electrophoresis. J. Chromatogr. A.

[29] Pérez-Fernández V. (2013). Separación Enantiomérica y/o Determinación de Compuestos de Interés Medioambiental por Metodologías Analíticas Electroforéticas y Cromatográficas Innovadoras. Ph.D. Thesis.

[30] Saz J.M., Marina M.L. (2016). Recent advances on the use of cyclodextrins in the chiral analysis of drugs by capillary electrophoresis. J. Chromatogr. A.

[31] Szejtli J. (1998). Introduction and general overview of cyclodextrin chemistry. Chem. Rev..

[32] Terabe S., Otsuka K., Ichikawa K., Tsuchiya A., Ando T. (1984). Electrokinetic separations with micellar solutions and open-tubular capillaries. Anal. Chem..

[33] Nishi H., Fukuyama T., Terabe S. (1991). Chiral separation by cyclodextrin-modified micellar electrokinetic chromatography. J. Chromatogr. A.

[34] Messina A., Sinibaldi M. (2007). CEC enantioseparations on chiral monolithic columns: A study of the stereoselective degradation of (R/S)-dichlorprop 2-(2,4-dichlorophenoxy)propionic acid in soil. Electrophoresis.

[35] Pérez-Fernández V., Dominguez-Vega E., Chankvetadze B., Grego A.L., García M.A., Marina M.L. (2012). Evaluation of new cellulose-based chiral stationary phases Sepapak-2 and Sepapak-4 for the enantiomeric separation of pesticides by nano liquid chromatography and capillary electrochromatography. J. Chromatogr. A.

[36] Huang L., Lin J., Xu L., Chen G. (2007). Nonaqueous and aqueous-organic media for the enantiomeric separations of neutral organophosphorus pesticides by CE. Electrophoresis.

[37] Polcaro C.M., Marra C., Desiderio C., Fanali S. (1999). Stereoselective analysis of acid herbicides in natural waters by capillary electrophoresis. Electrophoresis.

[38] Desiderio C., Polcaro C.M., Padiglioni P., Fanali S. (1997). Enantiomeric separation of acidic herbicides by capillary electrophoresis using vancomycin as chiral selector. J. Chromatogr. A.

[39] Naghdi E., Fakhari A.R., Ghasemi J.B. (2020). Enantioseparation and quantitative determination of ibuprofen using vancomycin-mediated capillary electrophoresis combined with molybdenum disulfide-assisted dispersive solid-phase extraction: Optimization using experimental design. J. Iran. Chem. Soc..

[40] Asami T., Imura H. (2006). Absolute determination method for trace quantities of enantiomer of glufosinate by gamma-cyclodextrin modified capillary zone electrophoresis combined with solid-phase extraction and on-capillary concentration. Anal. Sci..

[41] Klein C., Schneider R.J., Meyer M.T., Aga D.S. (2006). Enantiomeric separation of metolachlor and its metabolites using LC-MS and CZE. Chemosphere.

[42] Yi F., Guo B., Peng Z., Li H., Marriott P., Lin J.M. (2007). Study of the enantioseparation of imazaquin and enantioselective degradation in field soils by CZE. Electrophoresis.

[43] García-Cansino L., García M.A., Marina M.L. (2021). Simultaneous enantiomeric separation of carfentrazone-ethyl herbicide and its hydrolysis metabolite carfentrazone by cyclodextrin electrokinetic chromatography. Analysis of agrochemical products and a degradation study. Molecules.

[44] Wu Y.S., Lee H.K., Li S.F.Y. (2000). Simultaneous chiral separation of triadimefon and triadimenol by sulfated beta-cyclodextrin-mediated capillary electrophoresis. Electrophoresis.

[45] Ibrahim W.A.W., Hermawan D., Sanagi M.M. (2007). On-line preconcentration and chiral separation of propiconazole by cyclodextrin-modified micellar electrokinetic chromatography. J. Chromatogr. A.

[46] Jiménez-Jiménez S., Castro-Puyana M., Marina M.L., García M.Á. (2021). Enantiomeric separation of prothioconazole and prothioconazole-desthio by capillary electrophoresis. Degradation studies in environmental samples. J. Chromatogr. A.

[47] Chu B.L., Guo B.Y., Wang Z., Guo G., Lin J.M. (2007). Studies on degradation of imazalil enantiomers in soil using capillary ellectrophoresis. J. Sep. Sci..

[48] Kodama S., Yamamoto A., Ohura T., Matsunaga A., Kanbe Y. (2003). Enantioseparation of imazalil residue in orange by capillary electrophoresis with 2-hydroxypropyl-beta-cyclodextrin as a chiral selector. J. Agric. Food Chem..

[49] Lecoeur-Lorin M., Delepee R., Morin P. (2009). Simultaneous enantioselective determination of fenamiphos and its two metabolites in soil sample by CE. Electrophoresis.

[50] Lewis D.L., Garrison A.W., Wommack K.E., Whittemmore A., Steudler P., Melillo J. (1999). Influence of environmental changes on degradation of chiral pollutants in soils. Nature.

[51] Jarman J.L., Jones W.J., Howell L.A., Garrison A.W. (2005). Application of capillary electrophoresis to study the enantioselective transformation of five chiral pesticides in aerobic soil slurries. J. Agric. Food Chem..

[52] Garrison A.W., Schmitt P., Martens D., Kettrup A. (1996). Enantiomeric selectivity in the environmental degradation of dichlorprop as determined by high performance capillary electrophoresis. Environ. Sci. Technol..

[53] Valimaña-Traverso J., Morante-Zarcero S., Pérez-Quintanilla D., García M.A., Sierra I., Marina M.L. (2018). Periodic mesoporous organosilica materials as sorbents for solid-phase extraction of drugs prior to simultaneous enantiomeric separation by capillary electrophoresis. J. Chromatogr. A.

[54] García-Ruiz C., Álvarez-Llamas G., Puerta Á., Blanco E., Sanz-Mendel A., Marina M.L. (2005). Enantiomeric separation of organophosphorus pesticides by capillary electrophoresis-Application to the determination of malathion in water samples after preconcentration by off-line solid-phase extraction. Anal. Chim. Acta.

[55] Hsieh Y.Z., Huang H.Y. (1996). Analysis of chlorophenoxy acid herbicides by cyclodextrin-modified capillary electrophoresis. J. Chromatogr. A.

[56] Kodama S., Yamamoto A., Saitoh Y., Matsunaga A., Okamura K., Kizu R., Hayakawa K. (2002). Enantioseparation of vindozolin by gamma-cyclodextrin-modified micellar electrokinetic chromatography. J. Agric. Food Chem..

[57] Shea D., Penmetsa K.V., Leidy R.B. (1999). Enantiomeric and isomeric separation of pesticides by cyclodextrin-modified micellar electrokinetic chromatography. J. AOAC Int..

[58] Silva M., Morante-Zarcero S., Pérez-Quintanilla D., Marina M.L., Sierra I. (2017). Preconcentration of beta-blockers using functionalized ordered mesoporous silica as sorbent for SPE and their determination in waters by chiral CE. Electrophoresis.

[59] Silva M., Morante-Zarcero S., Pérez-Quintanilla D., Marina M.L., Sierra I. (2018). Environmental chiral analysis of beta-blockers: Evaluation of different n-alkyl-modified SBA-15 mesoporous silicas as sorbents in solid-phase extraction. Environ. Chem..

[60] Valimaña-Traverso J., Morante-Zarcero S., Pérez-Quintanilla D., García M.A., Sierra I., Marina M.L. (2018). Cationic amine-bridged periodic mesoporous organosilica materials for off-line solid-phase extraction of phenoxy acid herbicides from water samples prior to their simultaneous enantiomeric determination by capillary electrophoresis. J. Chromatogr. A.

[61] Petr J., Ginterová P., Znaleziona J., Knob R., Lošťáková M., Maier V., Ševčík J. (2013). Separation of ketoprofen enantiomers at nanomolar concentration levels by micellar electrokinetic chromatography with on-line electrokinetic preconcentration. Cent. Eur. J. Chem..

[62] Garrison A.W., Schmitt-Kopplin P., Avants J.K., Schmitt-Kopplin P. (2008). Analysis of the enantiomers of chiral pesticides and other pollutants in environmental samples by capillary electrophoresis. Methods in Molecular Biology.

[63] Pérez-Fernández V., García M.A., Marina M.L. (2010). Enantiomeric separation of *cis*-bifenthrin by CD-MEKC: Quantitative analysis in a commercial insecticide formulation. Electrophoresis.

[64] Pérez-Fernández V., García M.A., Marina M.L. (2011). Chiral separation of metalaxyl and benalaxyl fungicides by electrokinetic chromatography and determination of enantiomeric impurities. J. Chromatogr. A.

[65] Gupta R.C. (2019). Biomarkers in Toxicology.

[66] MAPA (2018). Encuesta de Comercialización de Productos Fitosanitarios.

[67] Insecticides. https://www.epa.gov/caddis-vol2/insecticides.

[68] O’Mahony T., Moore S., Brosnan B., Glennon J.D. (2003). Monitoring the supercritical fluid extraction of pyrethroid pesticides using capillary electrochromatography. Int. J. Environ. Anal. Chem..

[69] Liu T.L., Wang Y.S., Yen J.H. (2005). Separation of bifenthrin enantiomers by chiral HPLC and determination of their toxicity to aquatic organism. J. Food Drug Anal..

[70] Zhe X., Wenwei X., Hua H., Lirui P., Xu X. (2008). Direct chiral resolution and its application to the determination of the pesticide tetramethrin in soil by high-performance liquid chromatography using polysaccharide-type chiral stationary phase. J. Chromatogr. Sci..

[71] Eliel E.L., Wilen S.H. (1994). Stereochemistry of Organic Compounds.

[72] Morrissey C.A., Mineau P., Devries J.H., Sanchez-Bayo F., Liess M., Cavallaro M.C., Liber K. (2015). Neonicotinoid contamination of global surface waters and associated risk to aquatic invertebrates: A review. Environ. Int..

[73] Van den Brink P.J., Semeden J.M.V., Bekele R.S., Dierick W., De Gelder D.M., Noteboom M., Roessink I. (2016). Acute and chronic toxicity of neonicotinoids to nymphs of a mayfly species and some notes on seasonal differences. Environ. Toxicol. Chem..

[74] Hossain M. (2015). Recent perspective of herbicide: Review of demand and adoption in world agriculture. J. Bangladesh Agric. Univ..

[75] Tejedor A.S. La Industria Agroquímica. https://www.eii.uva.es/organica/qoi/tema-12.php.

[76] PubChem, National Library of Medicine https://pubchem.ncbi.nlm.nih.gov/compound/4794.

[77] Charles R. (2004). Modelling Pesticides Residues. Ph.D. Thesis.

[78] Willis G.H., McDowell L.L., Ware G.W. (1987). Pesticide persistence on foliage. Reviews of Environmental Contamination and Toxicology: Continuation of Residue Reviews.

[79] Garrison A.W., Avants J.K., Miller R.D. (2011). Loss of propiconazole and its four stereoisomers from the water phase of two soil-water slurries as measured by capillary electrophoresis. Int. J. Environ. Res. Public Health.

[80] The 2019 European Union Report on Pesticide Residues in Food. https://www.efsa.europa.eu/en/efsajournal/pub/6491.

[81] Ntalli N.G., Caboni P. (2012). Botanical Nematicides: A Review. J. Agric. Food Chem..

[82] Jackson R., Bellamy M. (2015). Antihypertensive drugs. BJA Educ..

[83] Nguyen L.A., He h Pham-Huy C. (2006). Chiral drugs: An overview. Int. J. Biomed. Sci..

[84] Godoy A.A., Kummrow P., Pamplin P.A.Z. (2015). Occurrence, ecotoxicological effects and risk assessment of antihypertensive pharmaceutical residues in the aquatic environment—A review. Chemosphere.

[85] De Oliveira A.R.M., Cesarino E.J., Bonato P.S. (2005). Solid-phase microextraction and chiral HPLC analysis of ibuprofen in urine. J. Chromatogr. B Anal. Technol. Biomed. Life Sci..

[86] Mai T.D., Bomastyk B., Duong H.A., Pham H.V., Hauser P.C. (2012). Automated capillary electrophoresis with on-line preconcentration by solid phase extraction using a sequential injection manifold and contactless conductivity detection. Anal. Chim. Acta.

[87] Mardones C., Ríos A., Valcárcel M. (2001). Determination of nonsteroidal anti-inflammatory drugs in biological fluids by automatic on-line integration of solid-phase extraction and capillary electrophoresis. Electrophoresis.

[88] Yilmaz B., Erdem A.F. (2014). Determination of ibuprofen in human plasma and urine by gas chromatography/mass spectrometry. J. AOAC Int..

[89] Commission Directive 93/67/EEC of 20 July 1993 Laying down the Principles for Assessment of Risks to Man and the Environment of Subtances Notified in Accordance with Council Directive 67/548/EEC. https://eur-lex.europa.eu/legal-content/EN/TXT/?uri=CELEX%3A31993L0067.

[90] García-Cansino L., Boltes K., Marina M.L., García M.A. (2023). Enantioseparation and ecotoxicity evaluation of ibrutinib by electrokinetic chromatography using single and dual systems. Talanta.

[91] Sanganyado E., Lu Z., Fu Q., Schlenk D., Gan J. (2017). Chiral pharmaceuticals: A review on their environmental occurrence and fate processes. Water Res..

[92] Casado N., Salgado A., Castro-Puyana M., García M.A., Marina M.L. (2019). Enantiomeric separation of ivabradine by cyclodextrin-electrokinetic chromatography. Effect of amino acid chiral ionic liquids. J. Chromatogr. A.

[93] Hrobonova K., Lomenova A. (2020). Determination of panthenol enantiomers in cosmetic preparations using an achiral-chiral-coupled column HPLC system. Chirality.

[94] Khater S., West C. (2015). Development and validation of a supercritical fluid chromatography method for the direct determination of enantiomeric purity of provitamin B5 in cosmetic formulations with mass spectrometric detection. J. Pharm. Biomed. Anal..

[95] Lomenova A., Hrobonova K., Solonyova T. (2018). HPLC separation of panthenol enantiomers on different types of chiral stationary phases. Acta Chim. Slovaca.

